# Author Correction: Hybridization is a recurrent evolutionary stimulus in wild yeast speciation

**DOI:** 10.1038/s41467-019-09702-z

**Published:** 2019-05-13

**Authors:** Chris Eberlein, Mathieu Hénault, Anna Fijarczyk, Guillaume Charron, Matteo Bouvier, Linda M. Kohn, James B. Anderson, Christian R. Landry

**Affiliations:** 1 PROTEO, The Quebec Network for Research on Protein Function, Engineering, and Applications, Québec, QC G1V 0A6 Canada; 2 Département de Biologie, Université Laval, Québec, QC G1V 0A6 Canada; 3 Institut de Biologie Intégrative et des Systèmes (IBIS), Université Laval, 1030 Ave de la Médecine, Québec, QC G1V 0A6 Canada; 4 Centre de recherche en données massives (CRDM), Université Laval, Québec, QC G1V 0A6 Canada; 5 Département de Biochimie, Microbiologie et Bio-informatique, Université Laval, Québec, QC G1V 0A6 Canada; 6 Departments of Ecology and Evolutionary Biology and Cell and Systems Biology, University of Toronto Mississauga, 3359 Mississauga Rd, Mississauga, ON L5L 1C6 Canada

**Keywords:** Evolutionary genetics, Speciation, Genetic hybridization

Correction to: *Nature Communications* 10.1038/s41467-019-08809-7, published online 25 February 2019

**Supplementary Information** accompanies this paper at 10.1038/s41467-019-09702-z.

The original version of the Supplementary Information associated with this Article contained errors in Supplementary Figures 2, 12, 20 and 22. The HTML has been updated to include a corrected version of the Supplementary Information; the original incorrect versions of these Figures can be found as Supplementary Information associated with this Correction.

## Supplementary information

Supplementary Information

