# Supplementary material for: Hybridization is a recurrent evolutionary stimulus in wild yeast speciation
Source: Nat Commun. 2019 Feb 25;10:923. doi: 10.1038/s41467-019-08809-7 (PMC6389940; doi:10.1038/s41467-019-08809-7)
Supplement: Supplementary file 1 — Supplementary Information [file 41467_2019_8809_MOESM1_ESM.pdf]

Supplementary Information

Hybridization is a recurrent evolutionary stimulus in wild yeast speciation

Eberlein, Hénault et al.

SUPPLEMENTARY FIGURES

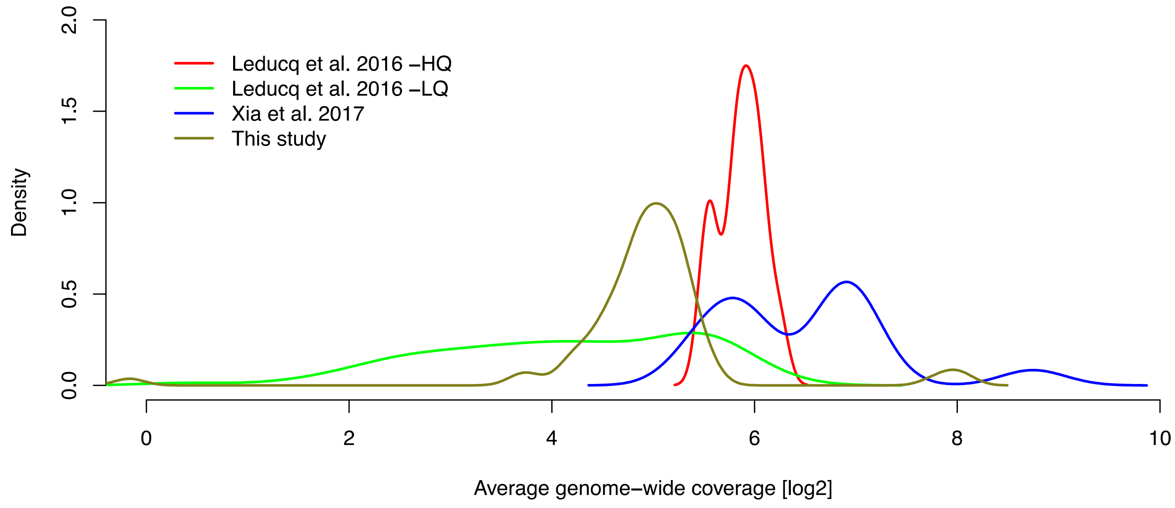

Supplementary Figure 1: Sequencing statistics of 316 genomes.
We aligned the reads of the initially 318 sequencing libraries, including 153 strains from Leducq et al.[1], 91 strains from Xia et al.[2] and 74 newly sequenced strains to the reference genome CBS432[3], which is the European *SpA* strain. Two strains were removed from the 74 newly sequenced genomes due to sequencing errors, making a final set of 316 sequences. The alignment rate for the 316 strains was on average 82 % and resulted in an average coverage depth of 54X per strain. The lowest coverage was observed for the *"low-quality"* genomes (LQ[1]), with an average of 24. The 72 newly sequenced genomes in this study showed an average genome-wide coverage of 40X.

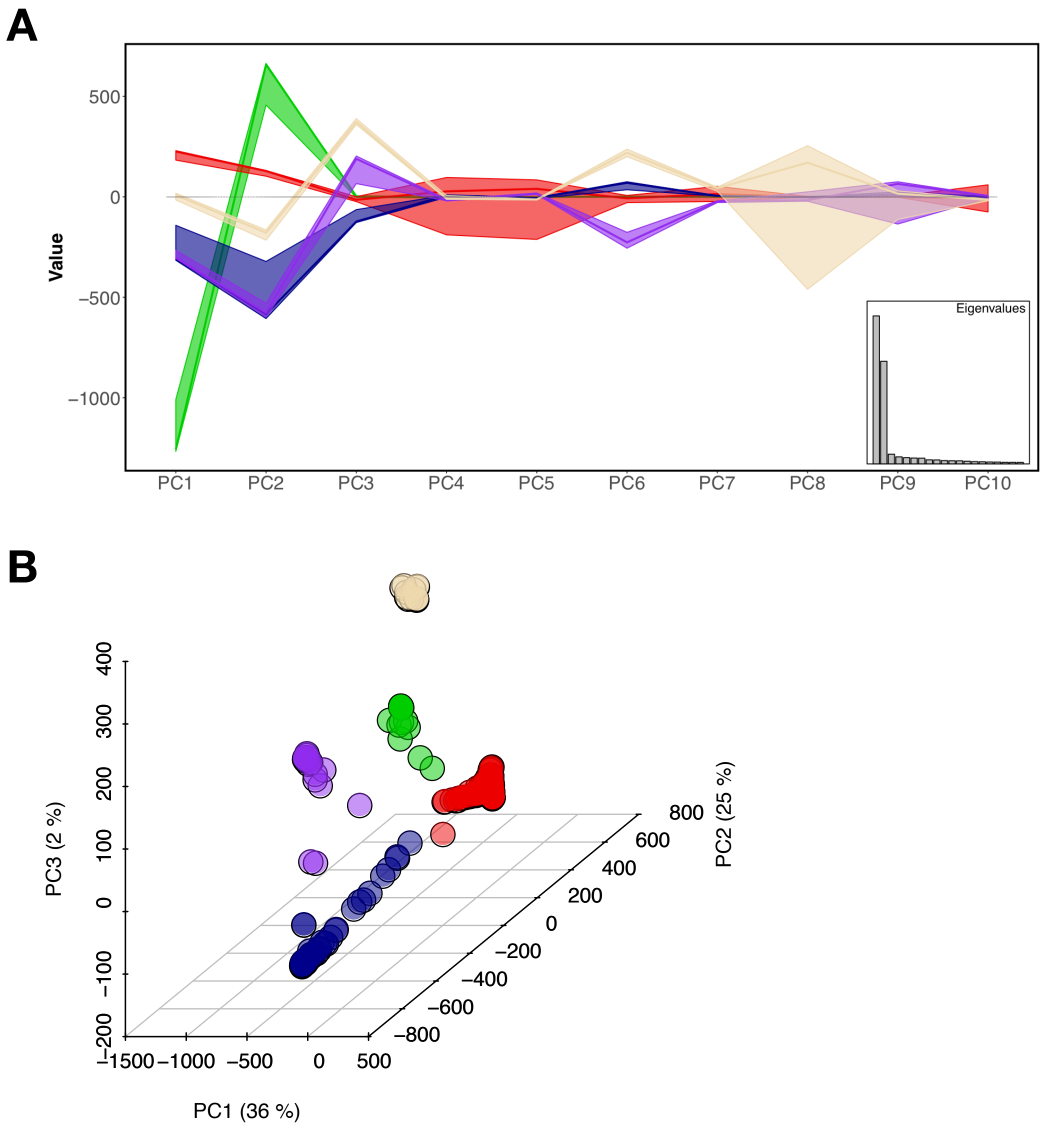

Supplementary Figure 2: Principal component analysis (PCA) from genome-wide SNP data distinguishes the 5 main *S. paradoxus* groups.
(A) A principal component analysis on 205,206 variants distributed genome-wide in 316 genomes (only showing the first ten PCs) was performed to examine the different *S. paradoxus* lineages. The bold line shows the median value for each lineage and shades the 15% and 85% quantiles. The lineages *SpC* and *SpC**, differentiate at PC3 and PC6. *SpD* clusters independently from the other lineages. **(B)** A 3D visualization of the first 3 PCs shows the separation into the 5 genetically different clusters. The colors correspond to the strains of the different lineages (green = *SpA*, red = *SpB*, blue = *SpC*, purple = *SpC**, beige = *SpD*).

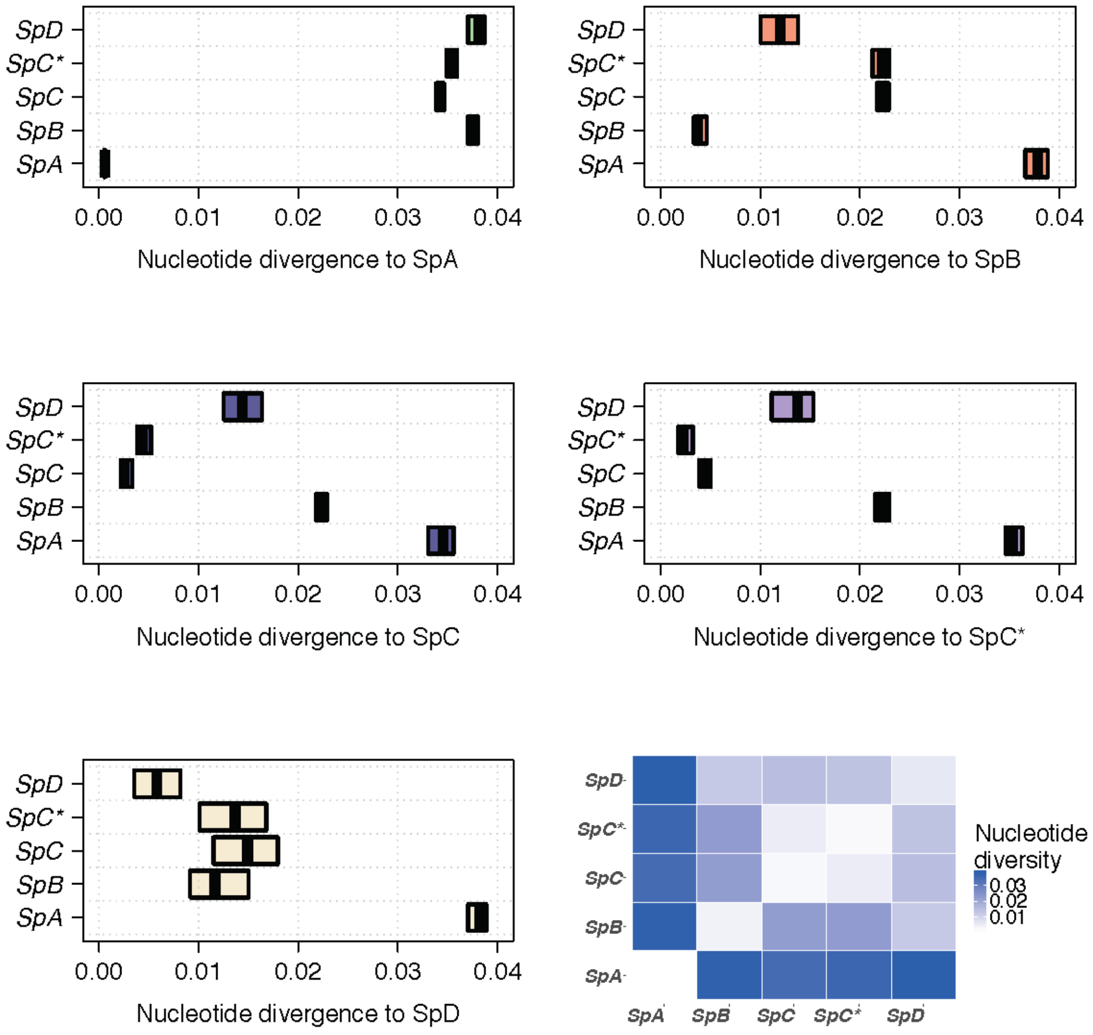

Supplementary Figure 3: Nucleotide diversity within and between lineages. Genetic diversity was assessed from genome-wide fasta-alignment files. The boxes represent the 25th and 75th percentiles with the median shown as a black bar. The colours of the bars correspond to the lineage was used as reference (green = *SpA*, red = *SpB*, blue = *SpC*, purple = *SpC**, beige = *SpD*).

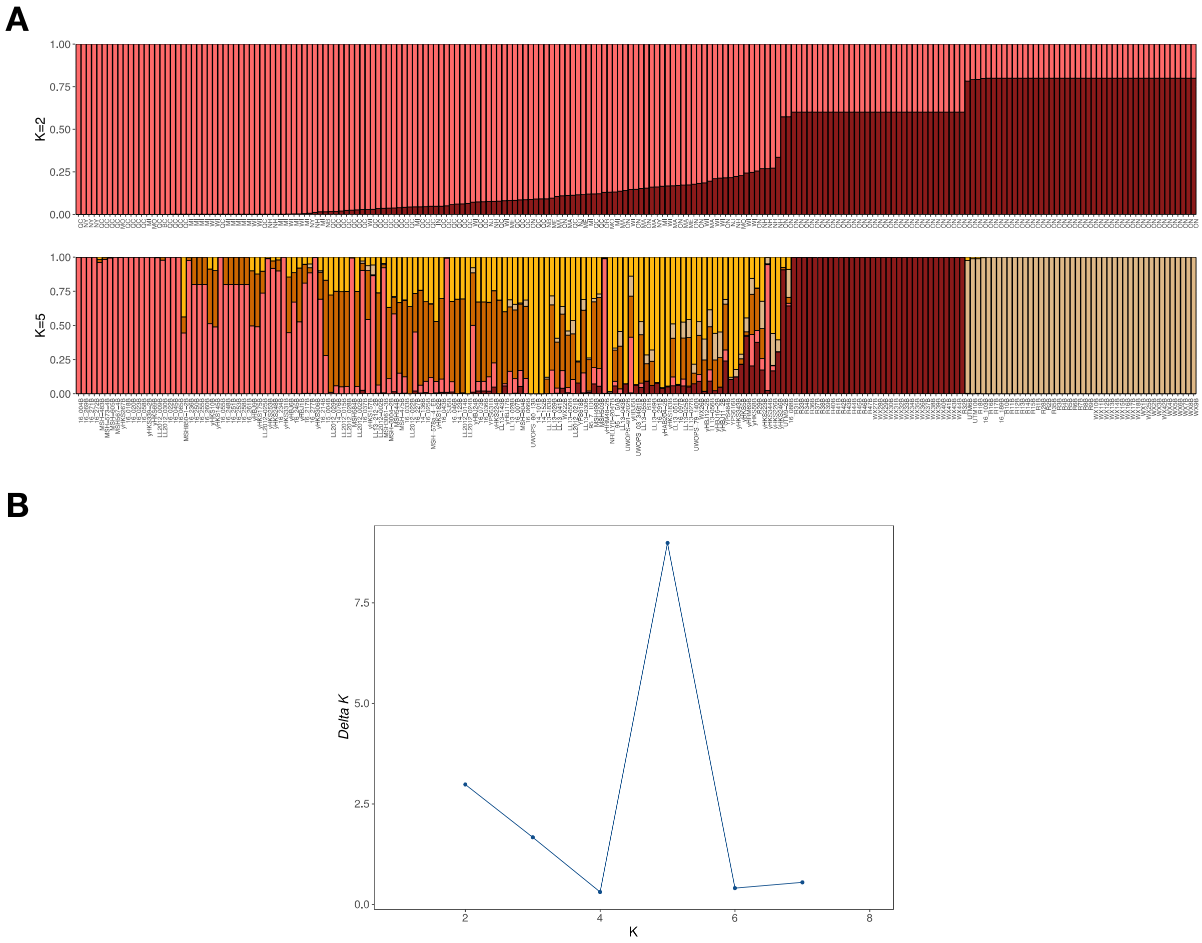

Supplementary Figure 4: Sub-population structure within the *SpB* lineage. (A) Bayesian inference of genetic structure in 213 *SpB* strains based on 7,973 filtered SNPs. The barplots show the proportion of membership of each *SpB* strain in different clusters. The two plots correspond to two clustering analyses assuming K = 2 and the most likely number of 5 clusters. Strains are ordered according to decreasing membership in the first cluster in the analysis assuming K = 2 clusters. The x-axis labels on the first plot are the abbreviations of the province/state names where the strains are located, and in the second plot they are the names of strains. This reveals potentially geographical population structure. **(B)** Plot of *Delta K* calculated with Harvester[4], as a function of K.

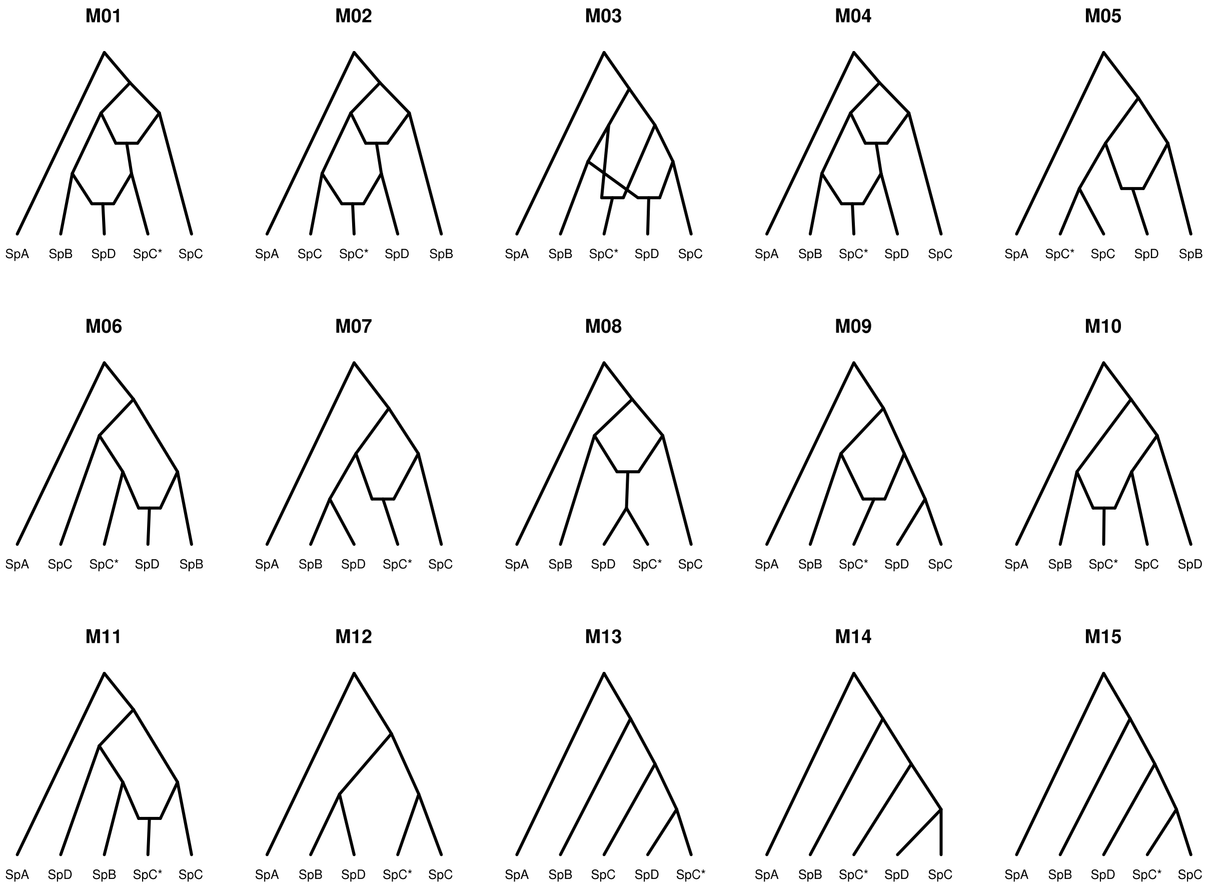

Supplementary Figure 5: 15 tested models examining the origin of *SpD* and *SpC.** Models assume admixed ancestry of *SpD* and *SpC** (M01, M02, M03, M04, M08), only admixed ancestry of *SpD* (M05, M06), only admixed ancestry of *SpC** (M07, M09, M10, M11), and no admixture between any lineages (M12, M13, M14, M15). Note that in model M03 the order of two admixture events is arbitrary.

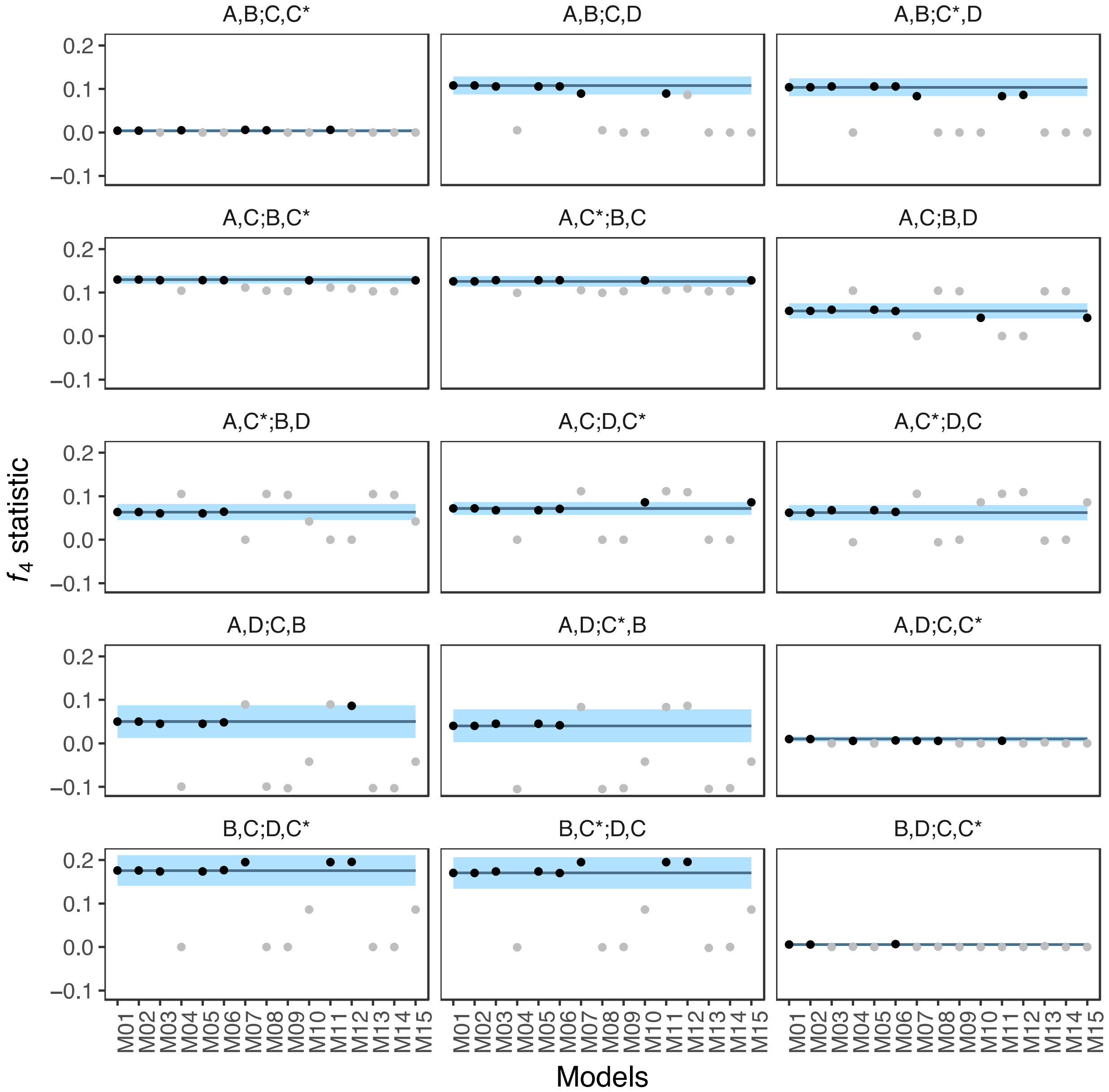

Supplementary Figure 6: The observed and the fitted values of *f4* statistics obtained for the 15 models.

f4 statistics were calculated in 15 combinations of unrooted topologies of 4 out of 5 lineages: A = *SpA*, B = *SpB*, C = *SpC*, C* = *SpC** and D = *SpD*. Blue horizontal line and the ribbon show the observed *f4* with the 3 standard errors, respectively. Black dots indicate model fitted values falling within the 3 standard errors of the observed values, and grey dots indicate fitted values falling outside of this range. Only the fitted values of models M01 and M02 match all the observed *f4*.

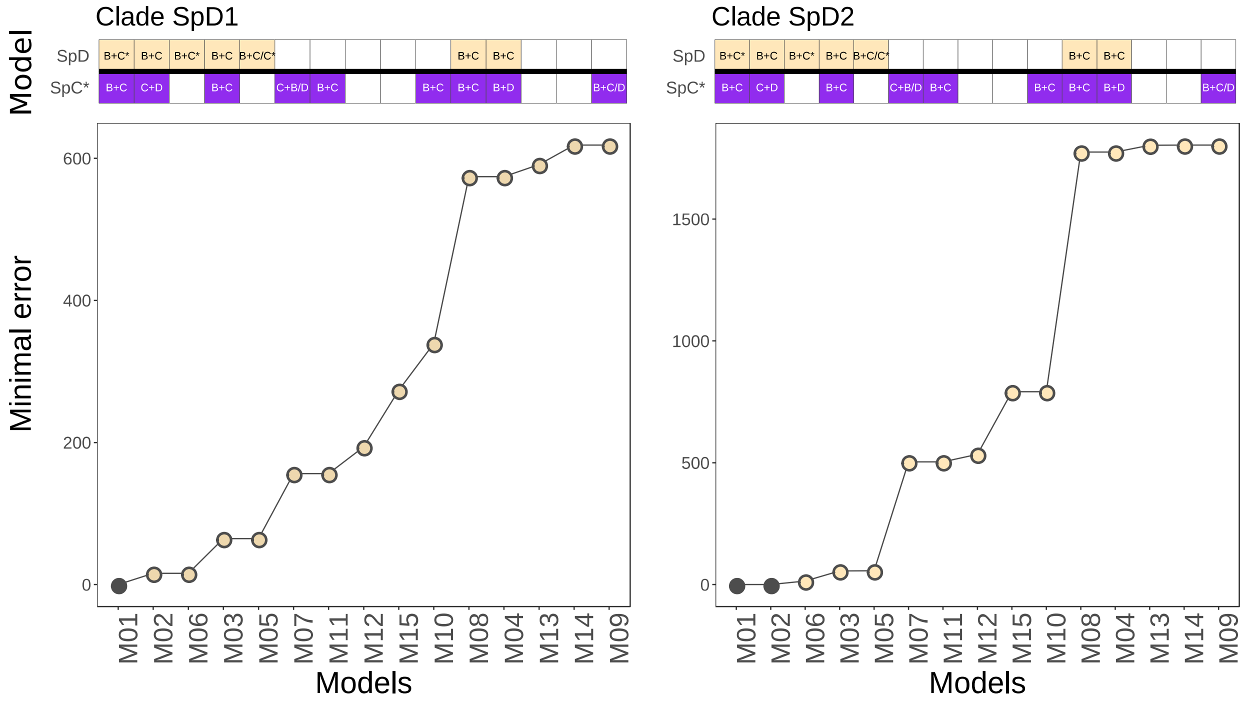

Supplementary Figure 7: Ranking of the 15 models based on their fit to the data, where only sequences from *SpD1* strains (left plot) or *SpD2* strains (right plot) were considered.

Filled squares on top indicate if the models assume admixed ancestry of *SpD1/SpD2* (yellow) and *SpC** (purple). Lineage abbreviations within the squares indicate hybrid parents. The two best models with fitted values within 3 standard errors of the observed summary statistics (filled dots), suggest hybrid origin of *SpD1/SpD2* resulting from a cross between *SpB* and the hybrid *SpC** (model M01), or hybrid origin of *SpD1/SpD2* resulting from a cross between *SpB* and *SpC*, occurring before the origin of a *SpC** hybrid (model M02). For both *SpD1* and *SpD2*, depending on the run, the model M02 did not always give a perfect match with the observed data.

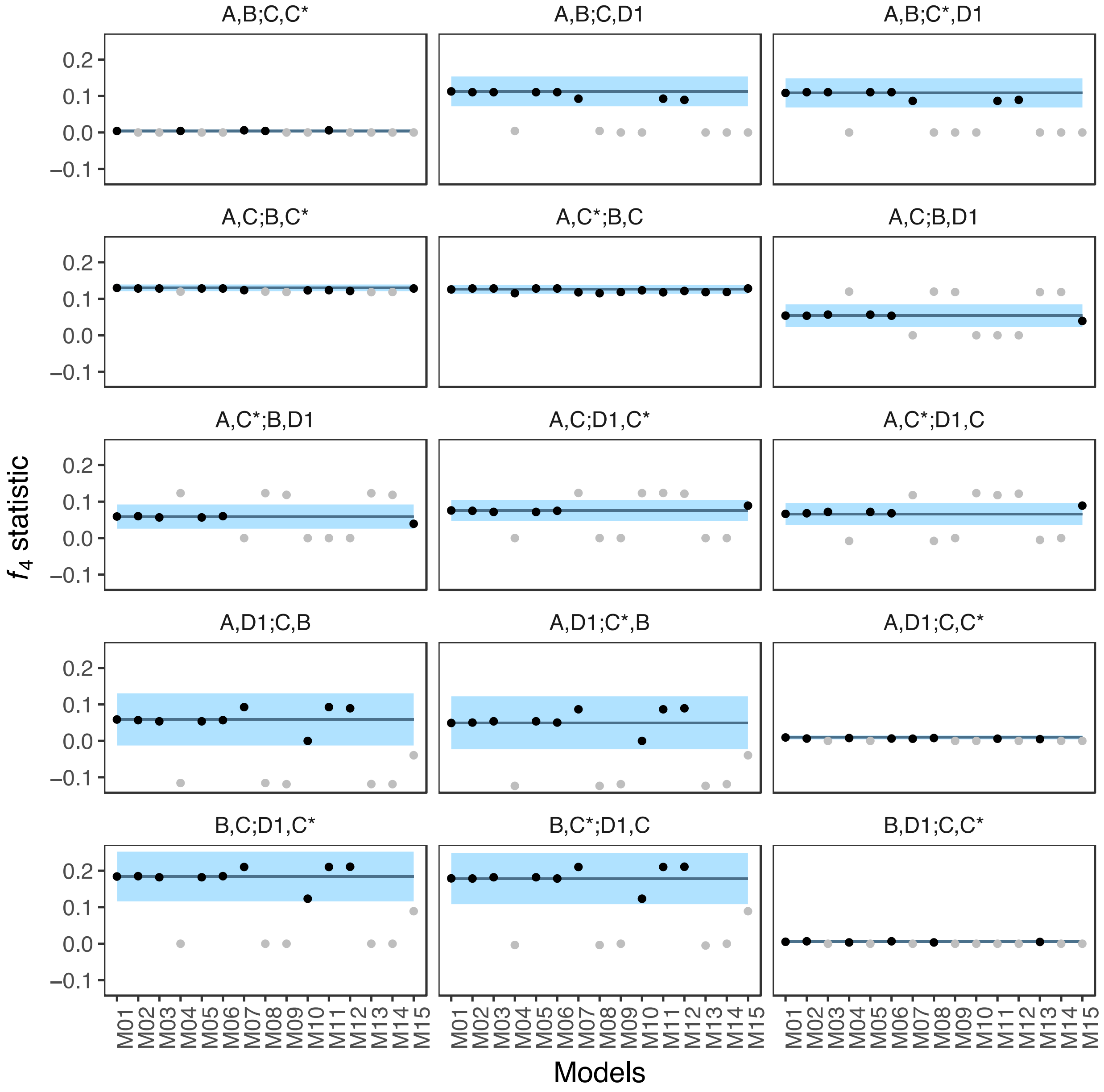

Supplementary Figure 8: The observed and the fitted values of *f4* statistics obtained for the 15 models with *SpD1* strains only.

Models were fitted to the data, where only strains of *SpD1* group were considered. *f4* statistics were calculated in 15 combinations of unrooted topologies of 4 out of 5 lineages: A = *SpA*, B = *SpB*, C = *SpC*, C* = *SpC** and D1 = *SpD1*. Blue horizontal lines and the ribbon show the observed *f4* with the 3 standard errors, respectively. Black dots indicate model fitted values falling within the 3 standard errors of the observed values, and grey dots indicate fitted values falling outside of this range. Only the fitted values of models M01 and M02 match all the observed *f4*.

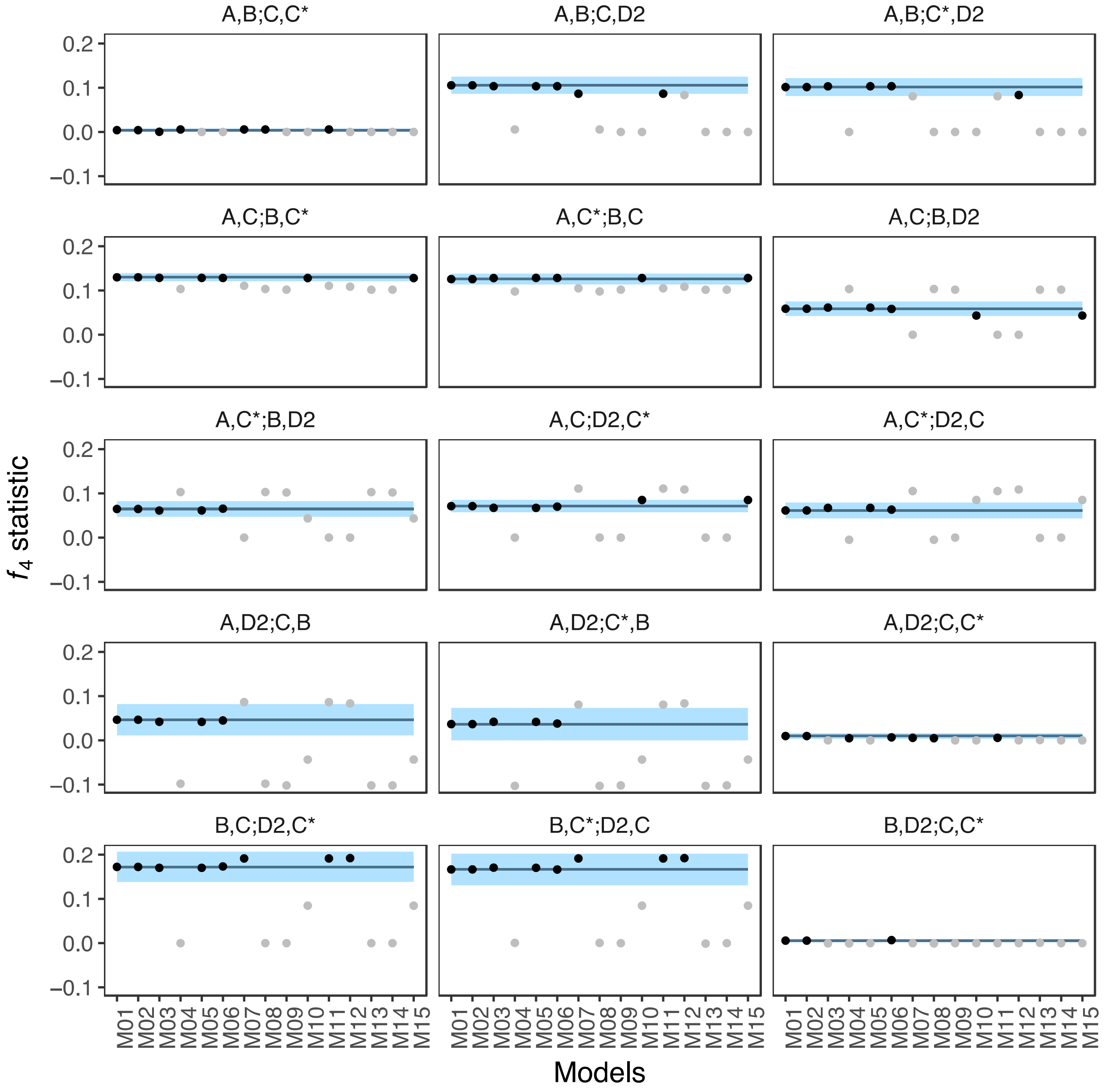

Supplementary Figure 9: The observed and the fitted values of *f4* statistics obtained for the 15 models with *SpD2* strains only.

Models were fitted to the data, where only strains of *SpD2* group were considered. *f4* statistics were calculated in 15 combinations of unrooted topologies of 4 out of 5 lineages: A = *SpA*, B = *SpB*, C = *SpC*, C* = *SpC** and D2 = *SpD2*. Blue horizontal line and the ribbon show the observed *f4* with the 3 standard errors, respectively. Black dots indicate model fitted values falling within the 3 standard errors of the observed values, and grey dots indicate fitted values falling outside of this range. Only the fitted values of models M01 and M02 match all the observed *f4*.

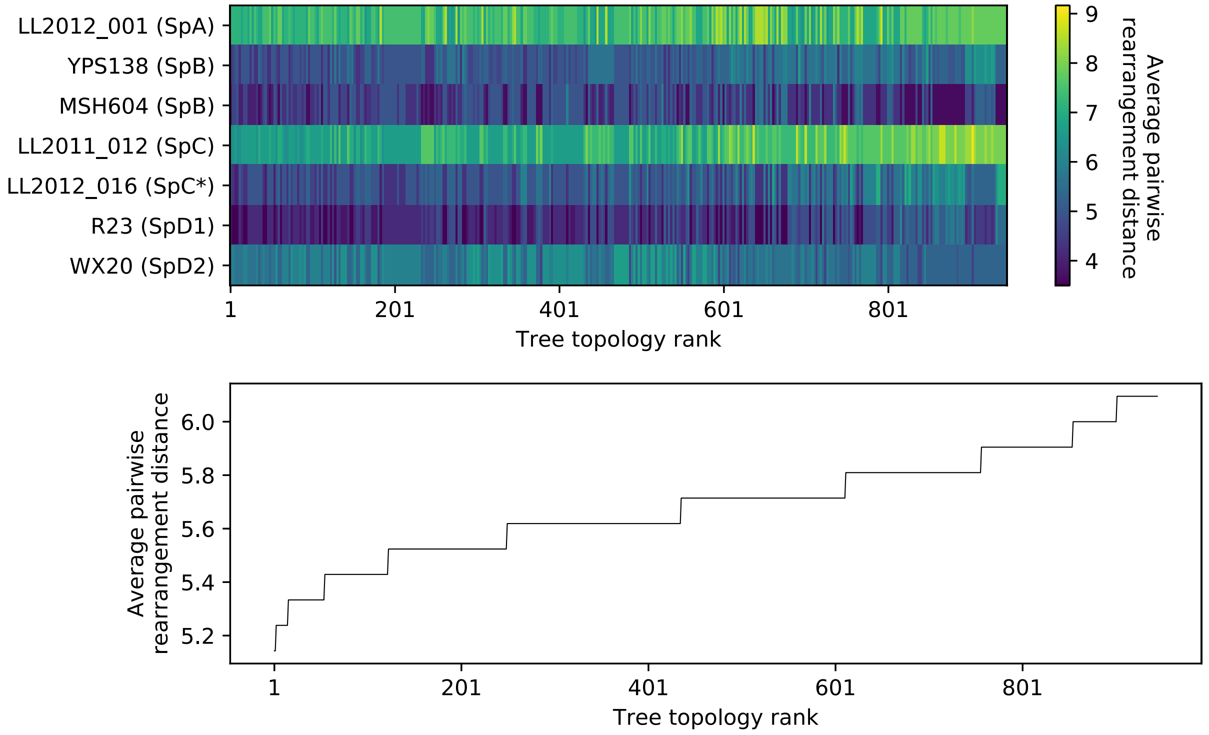

Supplementary Figure 10: Selection of the best tree topologies describing genomic rearrangements using a maximal parsimony criterion. Average pairwise rearrangement distance (in number of rearrangements) is displayed for individual terminal nodes (top) or for all terminal nodes aggregated (bottom). The topologies are ranked according to the aggregated average pairwise rearrangement distance.

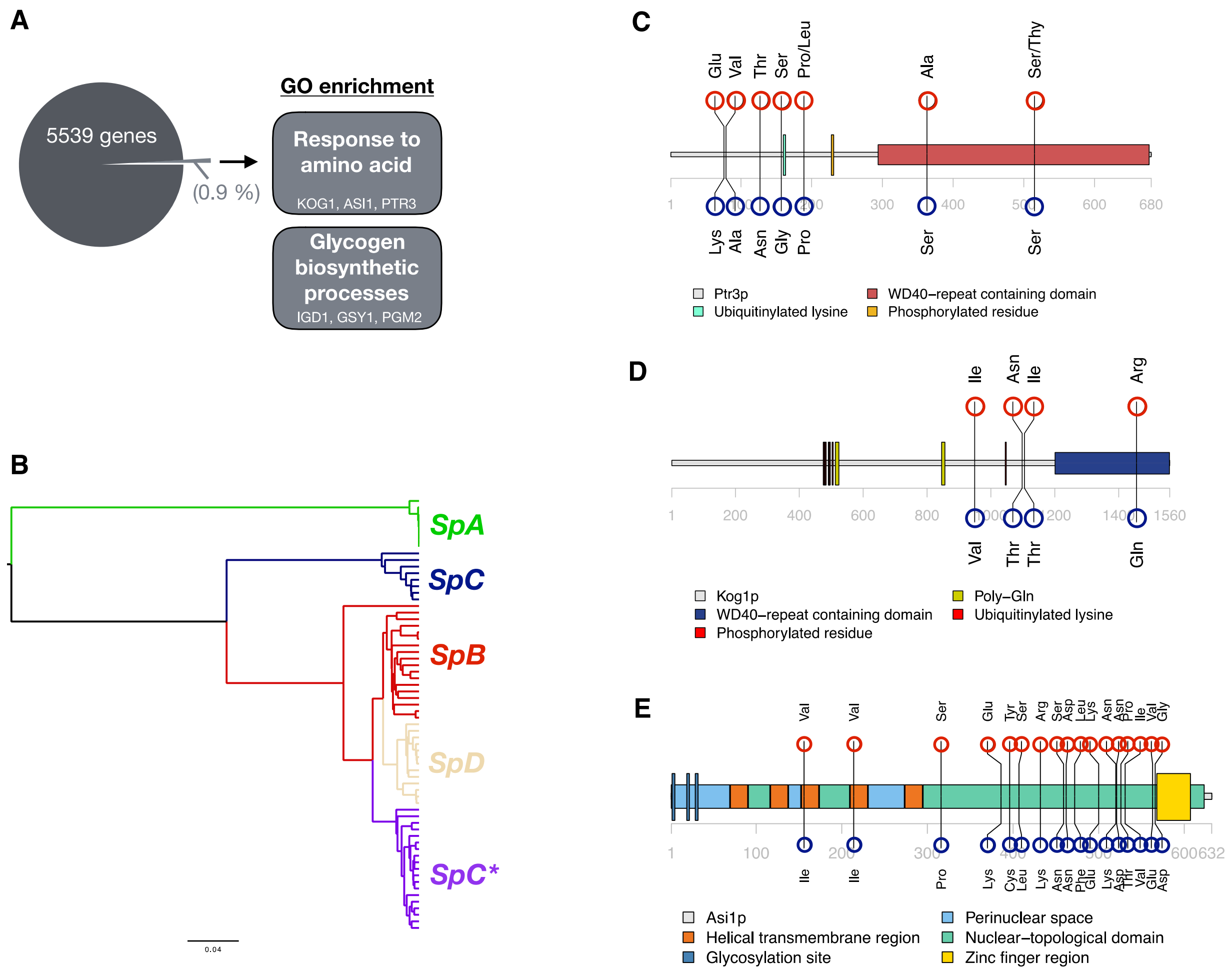

Supplementary Figure 11: Genes in *SpC introgressed from *SpB* are enrichment for the biological process *response to amino acid.***

(A) The 51 introgressed and fixed genes in *SpC** (*SpB*-like) revealed GO enrichment for response to amino acid and glycogen biosynthetic process (See: Supplementary table 8-9). **(B)** Phylogenetic tree showing the similarity of *SpB* introgression in *SpC**. Sequence similarity of 51 concatenated genes shows the inheritance from *SpB*. The new lineage *SpD* groups in-between *SpB* and *SpC**. The phylogenetic tree was constructed from a subset of 66 individuals. **(C-E)** Amino acid differences between *SpB* and *SpC* in the introgressed genes *PTR3*, *KOG1* and *ASI1.* These fixed amino acid changes in the introgressed genes *PTR3*, *KOG1* and *ASI1* could potentially have functional consequences (red circles: amino acids in *SpB*, blue circle: amino acid in *SpC*). Information about functional domains was obtained from http://www.rcsb.org/.

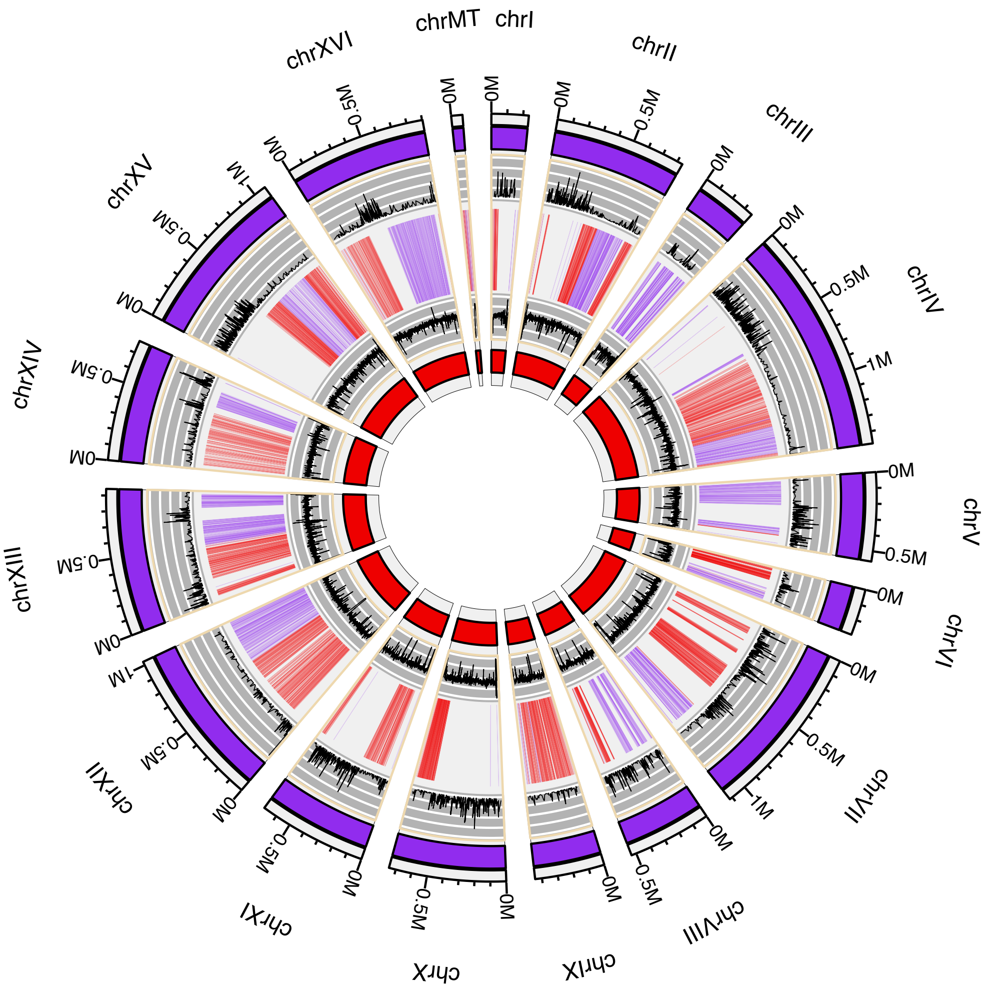

Supplementary Figure 12: *SpD* strain WX21 shows heterozygous loci across the genome.

The *SpD* strain WX21 shows many regions that could not be assigned confidently to *SpB* (inner red ring) or *SpC** (outer purple ring) introgression (blank regions, middle circle), which stands in contrast to the other 12 *SpD* strains (Figure 3A). We confirmed the presence of mapped sequence data (inner circle, coverage data corresponds to the average coverage from 1 kb windows) throughout the genome and could show that the non-defined regions (in white) correspond to peaks of heterozygosity (outer rings; counts of heterozygous sites were calculated per 1kb window).

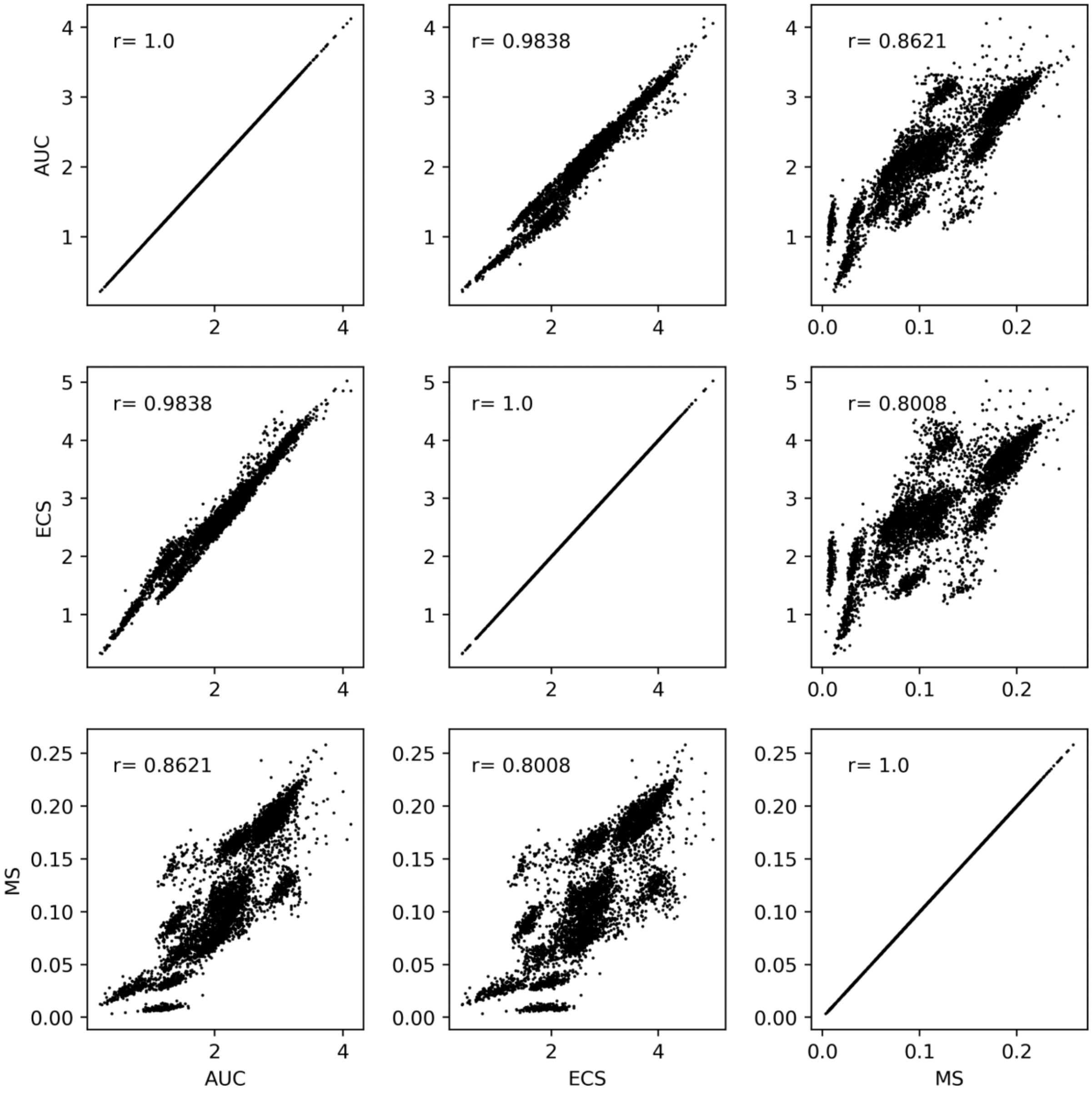

Supplementary Figure 13: Correlations between colony growth traits AUC, ECS and MS.

AUC corresponds to the integrated growth through time, ECS corresponds to the latest point of the growth curve, and MS corresponds to the maximal growth rate. See Methods for the calculation details for each trait.

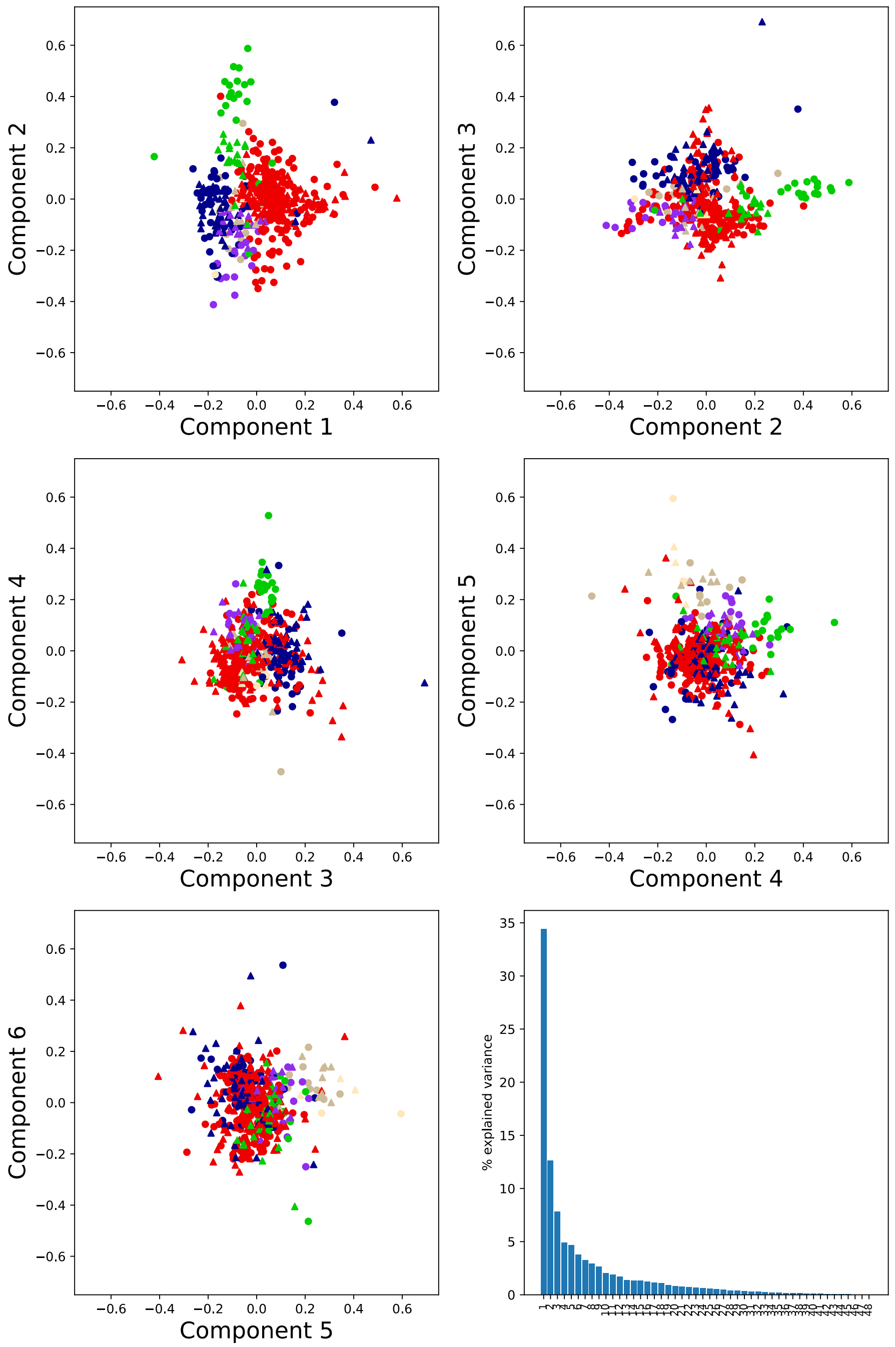

Supplementary Figure 14: Multiple factor analysis (MFA) performed on combined AUC and MS values.

The first six principal components are shown. Circles and triangles correspond to AUC and MS values, respectively. Dot colors correspond to the lineages where strains are color-coded (green = *SpA*, red = *SpB*, blue = *SpC*, purple = *SpC**, light beige = *SpD1*, dark beige = *SpD2*). The proportion of explained variance for each component is indicated above the corresponding bar in the bottom right subpanels.

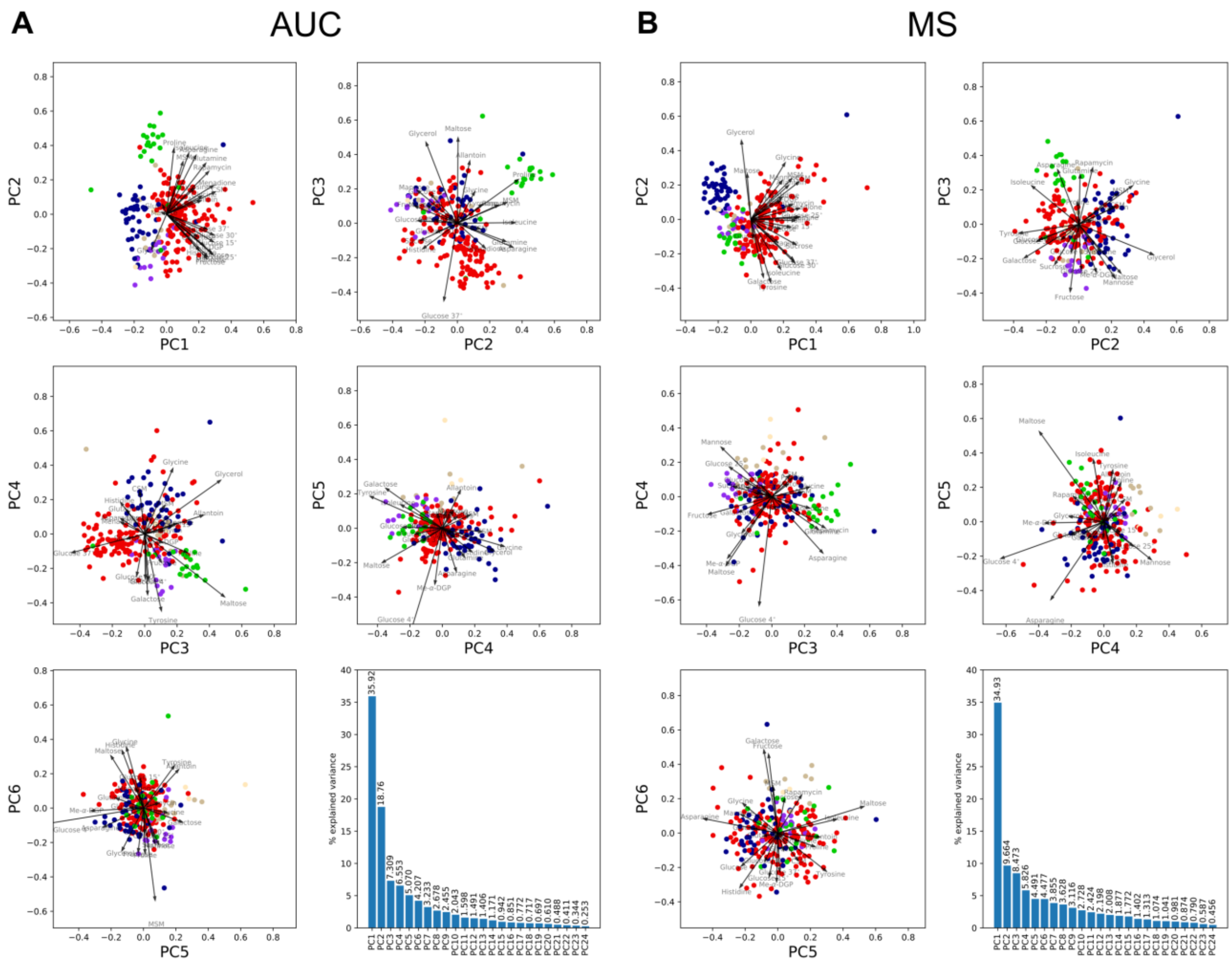

Supplementary Figure 15: Principal component analysis (PCA) performed on (A) AUC and (B) MS values.

The first six principal components are shown. Dot colors correspond to the lineages where strains are color-coded (green = *SpA*, red = *SpB*, blue = *SpC*, purple = *SpC**, light beige = *SpD1*, dark beige = *SpD2*). The proportion of explained variance for each principal component is indicated above the corresponding bar in the bottom right subpanels.

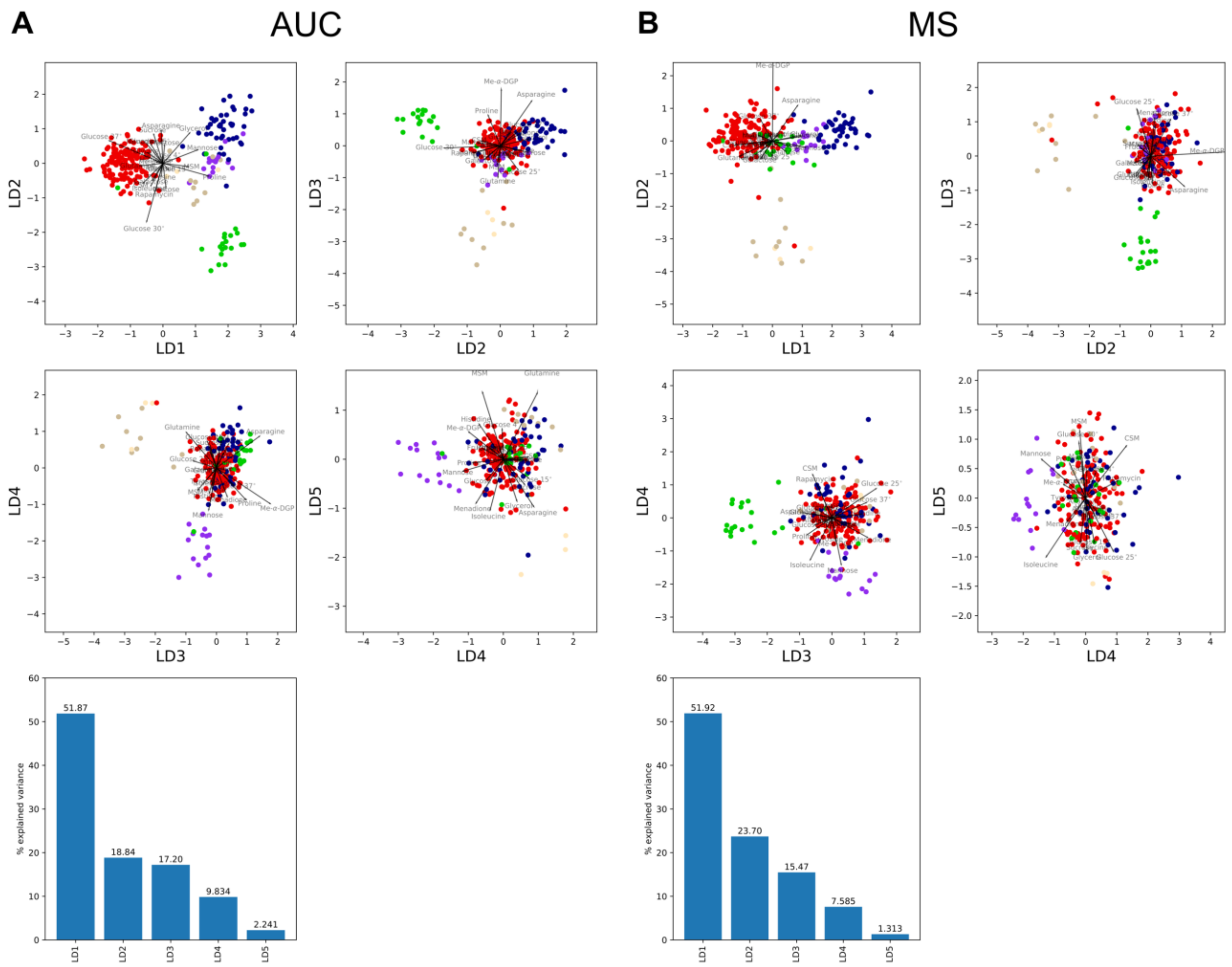

Supplementary Figure 16: Linear discriminant analysis (LDA) performed on (A) AUC and (B) MS values.

Dot colors correspond to the lineage where strains are color-coded (green = *SpA*, red = *SpB*, blue = *SpC*, purple = *SpC**, light beige = *SpD1*, dark beige = *SpD2*). The proportion of explained variance for each linear discriminant is indicated above the corresponding bar in the bottom right subpanels.

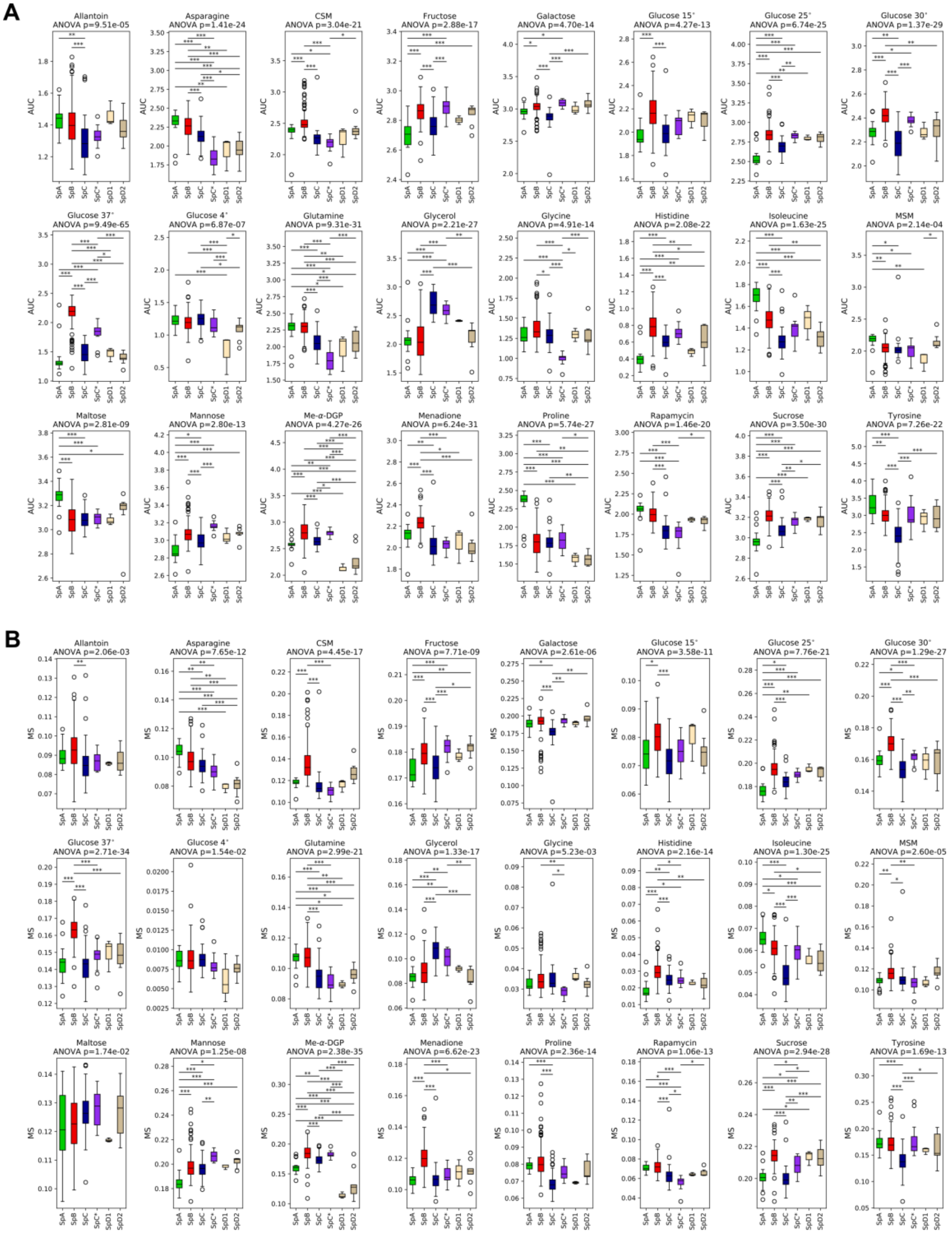

Supplementary Figure 17: Growth comparison per lineage for the (A) AUC and (B) MS traits.

Significant differences between lineages are indicated above the corresponding boxes (Tukey's HSD; p<0.05: *, p<0.01: **, p<0.001: ***). The p-value for one-way ANOVAs performed on data for each condition separately is shown. CSM: complete synthetic medium; Me-α-DGP: methyl-α-D-glucopyranoside; MSM:

minimal synthetic medium. The boxes represent the 25th and 75th percentiles with the median shown as a black bar.

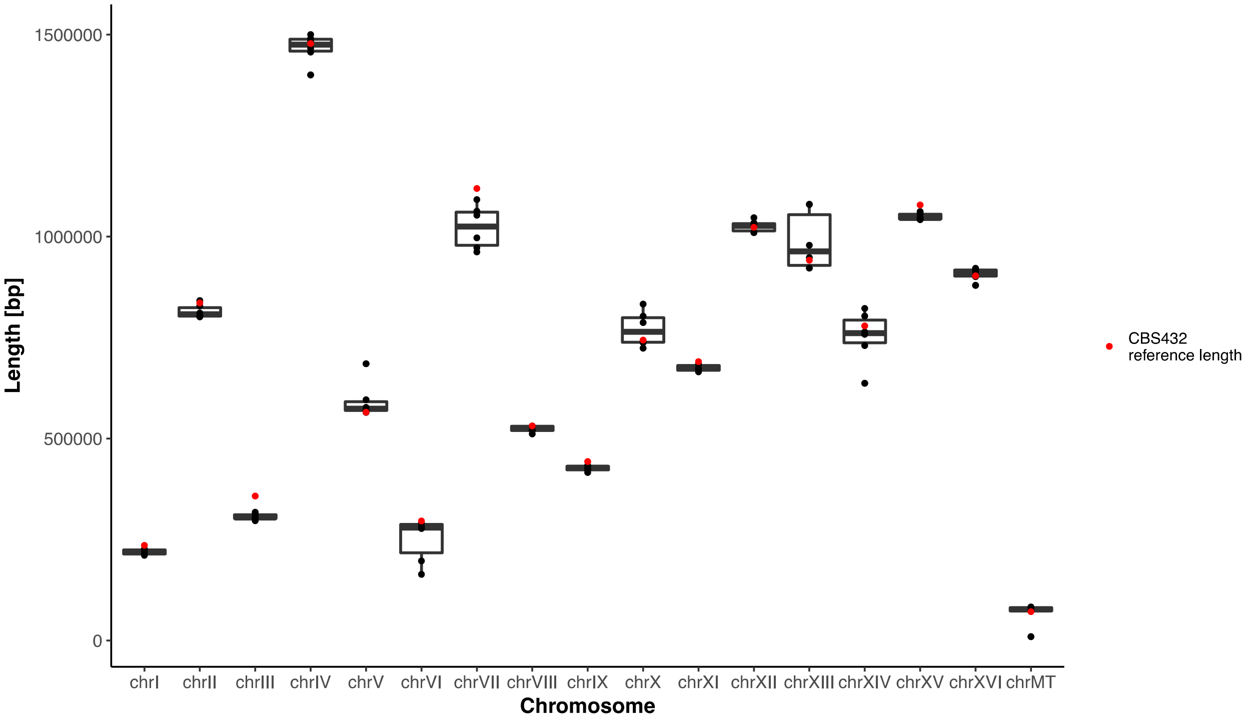

Supplementary Figure 18: *De novo* assembly of lineage-specific reference genomes for mapping genome-wide expression data.

We prepared reference genomes specific for the lineages *SpA, SpB, SpC, SpC** and one for each subclade of *SpD* (*SpD1, SpD2*). The 6 reference genomes show small variation in chromosome size among each other and to the reference genome CBS432[3]. The boxes represent the 25th and 75th percentiles with the median shown as a black bar.

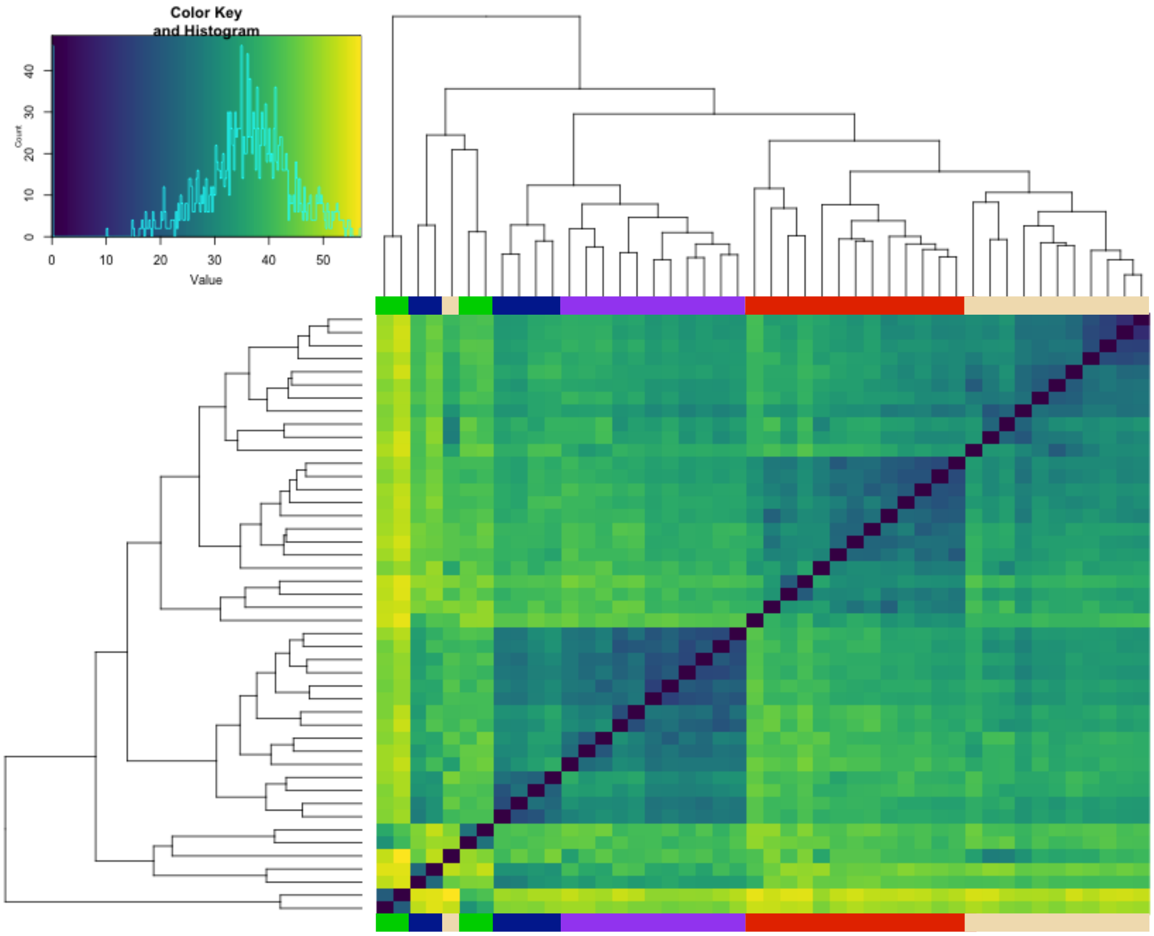

Supplementary Figure 19: Average genome-wide expression profile similarity for 46 strains from all main lineages of *S. paradoxus*.

Overall genome-wide expression calculated in DeSeq[5] from 5,160 genes showed similar relationships between the lineages as observed from genome-wide sequence data (Figure 1B-C). There is one outlier strain of *SpD*, which groups to *SpC*, however do not show any aberration in the principal component analyses (Figure 4D, Supplementary Figure 21). Strains are color-coded to distinguish lineages (green = *SpA*, red = *SpB*, blue = *SpC*, purple = *SpC**, beige = *SpD*).

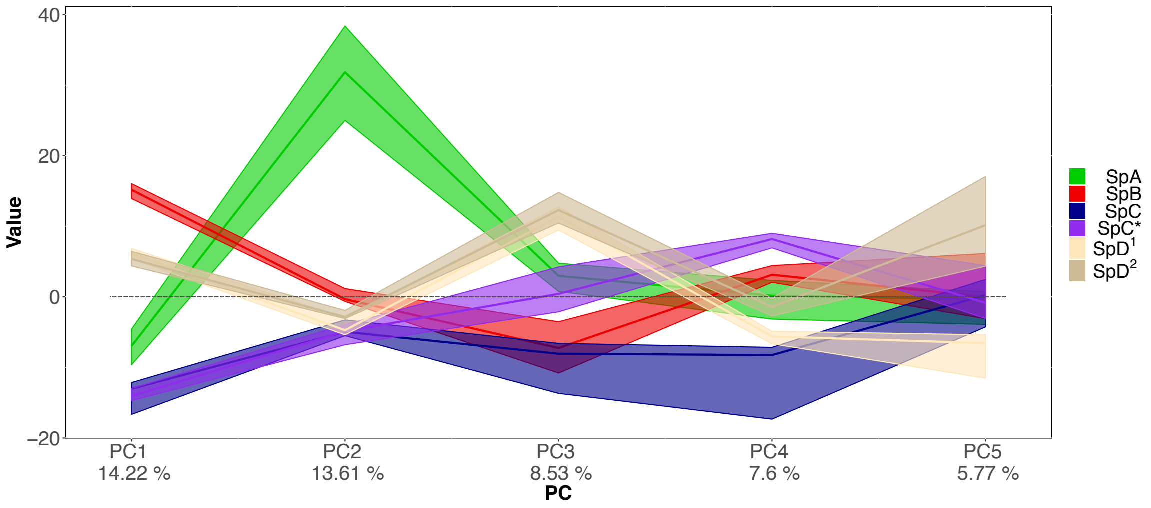

Supplementary Figure 20: Grouping of strains according to principal component analysis based on expression levels of 5,160 genes.
The most important PC, PC1, distinguishes the main groups *SpA*, *SpB*, *SpC* and *SpD* but does not distinguish *SpC** and *SpC*. PC3 and PC4 splits *SpC* and *SpC** strains into separate clusters, PC5 splits the two different *SpD* clades. Bold lines correspond to the median value and the color shades to the 15% and 85% quantiles.

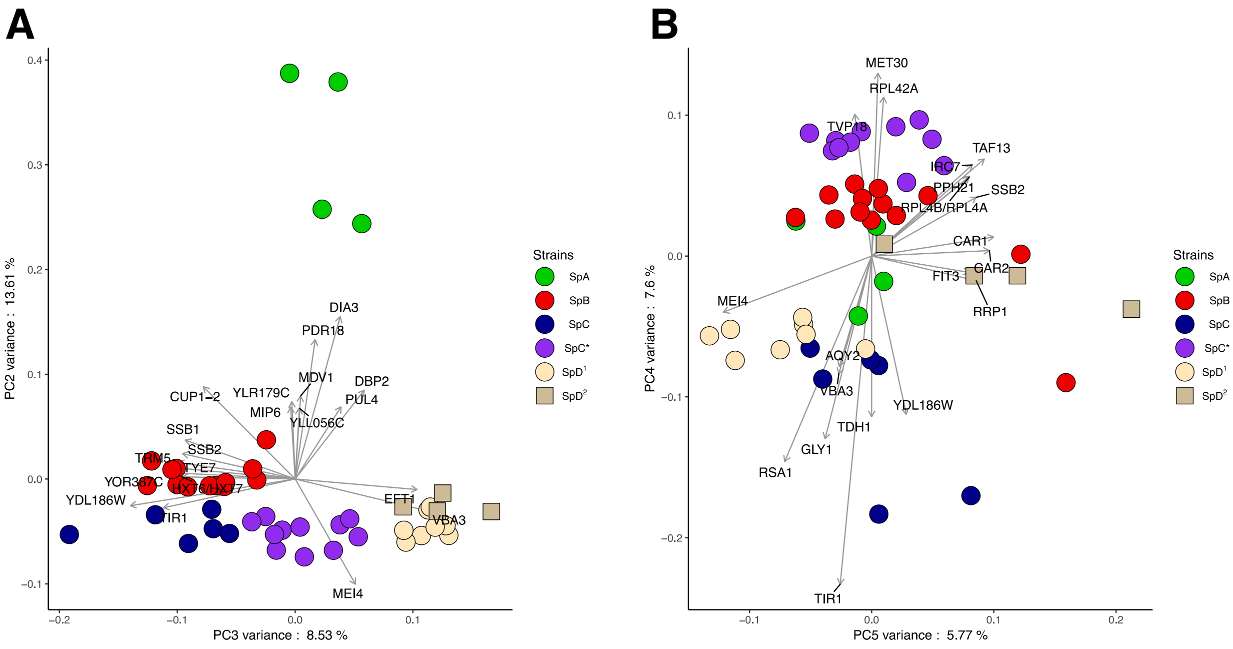

Supplementary Figure 21: Principal component analysis on genome-wide expression data splits the main lineages into independent clusters.

(A) Independent clusters for the lineages *SpC* and *SpC*.* The principal component PC3 explaining 8.5% of the variance separates the two lineages *SpC* and *SpC**. **(B)** PC4 and PC5, explaining cumulatively 13.4% of variance, splits the *SpD* lineage into the two subclades of *SpD1* and *SpD2.* Genes were ranked accordingly to their explained variance (high to low) and plots show only the 20 strongest candidates.

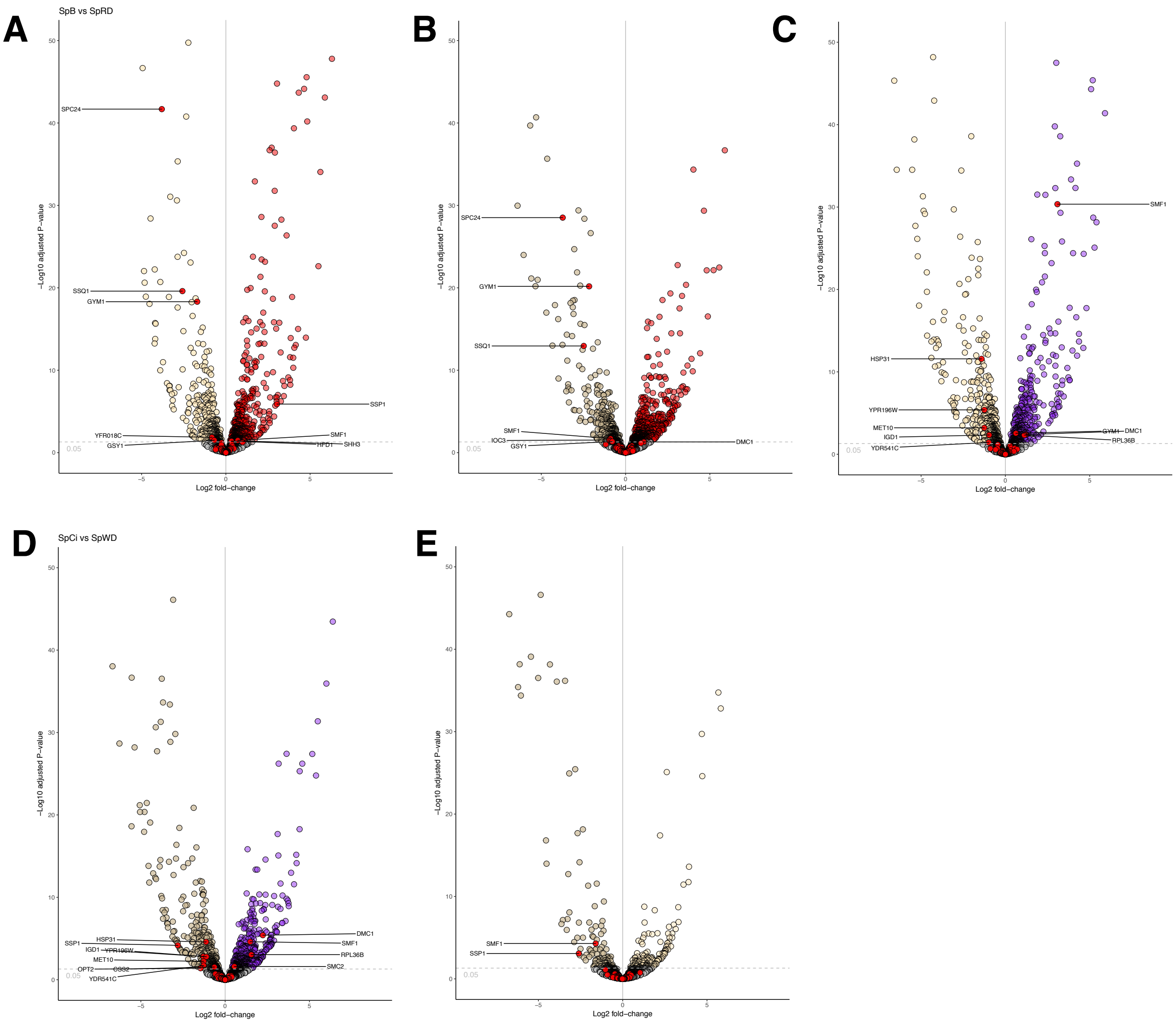

Supplementary Figure 22: Pairwise comparison of gene expression between the lineages *SpB*, *SpC*, *SpD1* and *SpD2*.

Pairwise comparison of **(A)** *SpB* and *SpD1,* **(B)** *SpB* and *SpD2,* **(C)** *SpC** and *SpD1,* **(D)** *SpC** and *SpD2,* and **(E)** *SpD1* and *SpD2.* Volcano plots showing the pairwise comparison of expression of 5,160 genes. We used the average expression per gene for each lineage to performed subsequent Gene-Ontology enrichment analyses on ranked data (p-adjust value; Supplementary table 7). Lineages are color coded (*SpB* = red, *SpC* = blue, *SpC** = purple, *SpD*= beige). In dark red are the expression profiles of 48 out of the 51 introgressed genes that are fixed in all *SpC** individuals and have been inherited from *SpB*.

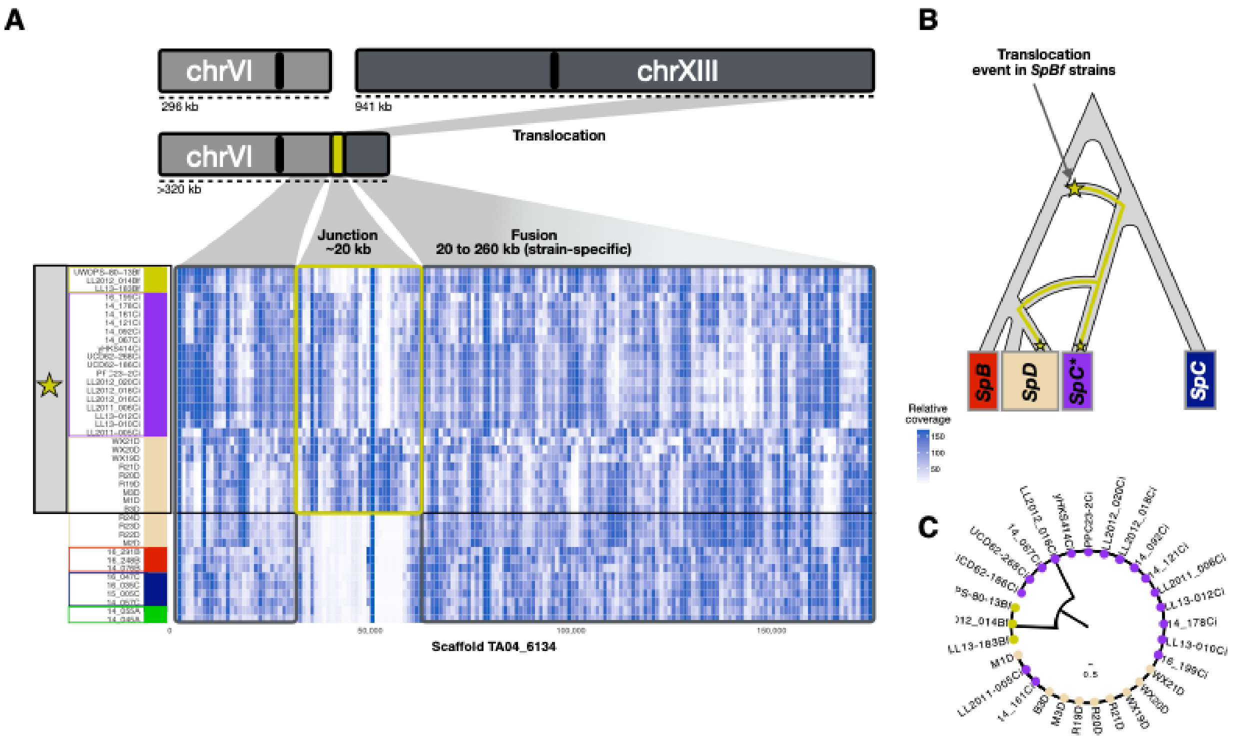

Supplementary Figure 23: Detection of genomic translocation VItXIII in a subset of 42 strains.

(A) *SpD2* inherited the translocation VItXIII. We detected the translocation VItXIII in *SpD* strains that belong to the sub-group *SpD2.* This translocation was previously identified in *SpC** and in *SpBf*. Using the scaffold TA04_6134 (~170 kb; *SpC** strain LL2012_018) from Leducq et al.[1], a fragment of ~20 kb was only present in the strains that contain the fusion of chromosome VI with the right arm of chromosome XIII. The fused part from chromosome XIII was shown to be variable in size, ranking from 20 up to 260 kb in different *SpC** strains[1]. The heat map represents the coverage along the scaffold calculated in 1 kb windows. **(B)** Schematic view the inheritance of VItXIII. Translocation VItXIII was transmitted from *SpBf* strains (3 *SpB* strains that carry the fusion; Leducq et al.[1]) into *SpC** upon secondary contact with *SpC*. It was further inherited only from *SpD2* from crosses between *SpC** and *SpB.* **(C)** Phylogenetic relationship of strains that carry the fusion. The ~20 kb fused fragment that corresponds to the junction sequence shows that *SpBf* strains are the sister clade to *SpC** and *SpD*, showing that this region is closer to *SpC** in *SpD* than it is to *SpB* strains.

SUPPLEMENTARY TABLES

Supplementary Table 1: Strains sequenced with long-read sequencing and de novo assembly statistics.

Systematic Name	Ploidy	Genotype	Genetic_Group	Location_Sampled	Substrate_Sampled
LL2012_001	Diploid	Wild	*SpA*	Hull, Quebec, Canada	Bark - Oak tree
MSH-604	Diploid	Wild	*SpB*	Mont St Hilaire, Quebec, Canada	Bark - Oak tree
LL2011_012	Diploid	Wild	*SpC*	Station Dushesnay, Quebec, Canada	Bark - Mapple tree
LL2012_016	Diploid	Wild	*SpC**	Pointe Platon, Quebec, Canada	Bark - Oak tree
R23	Diploid	Wild	*SpD* (*SpD1*)	University of Toronto Mississauga, Ontario, Canada	Quercus
WX20	Diploid	Wild	*SpD* (*SpD2*)	University of Toronto Mississauga, Ontario, Canada	Quercus

Systematic Name	Published in	lon	lat	Total size (nt)	Number of contigs	Number of chromosomes	N50 (nt)
LL2012_001	Leducq, et al. [1]	-75,7133658	45,428731	11861949	18	16	821342
MSH-604	Leducq, et al. [1]	-73,1790126	45,5640416	11835292	18	16	763954
LL2011_012	Leducq, et al. [1]	-71,6415386	46,870113	11959616	19	16	684142
LL2012_016	Leducq, et al. [1]	-71,859351	46,665643	12014617	19	16	692747
R23	Xia, et al. [2]	-79,6660259	43,5471097	11960863	20	16	778202
WX20	Xia, et al. [2]	-79,6660259	43,5471097	12028897	19	16	777535

Supplementary Table 2. Fifty-one fixed and introgressed genes in *SpC with *SpB* origin.**

Chromo-some	Gene Start [bp position]	Gene End [bp position]	Gene ID	Gene Name	Strand
chrMT	8430	14712	COB	COB	+
chrIII	106374	108275	CBS432_03G00420	YCL025C	-
chrIV	1459391	1460548	CBS432_04G07050	YDR532C	-
chrIV	1461318	1462031	CBS432_04G07060	YDR533C	-
chrIV	1474613	1475647	CBS432_04G07110	YDR541C	-
chrV	162251	163807	CBS432_05G00760	YER007W	+
chrV	165581	169597	CBS432_05G00780	YER008C	-
chrV	537305	538567	CBS432_05G02520	YER178W	+
chrV	538947	540042	CBS432_05G02530	YER179W	+
chrV	543277	543990	CBS432_05G02560	YER182W	+
chrVI	181326	181838	CBS432_06G00710	YFR011C	-
chrVI	182464	183885	CBS432_06G00720	YFR012W	+
chrVI	184488	186863	CBS432_06G00730	YFR013W	+
chrVI	187123	188460	CBS432_06G00740	YFR014C	-
chrVI	188845	190971	CBS432_06G00750	YFR015C	-
chrVI	191625	195269	CBS432_06G00760	YFR016C	-
chrVI	196339	196926	CBS432_06G00770	YFR017C	-
chrVI	197223	198308	CBS432_06G00780	YFR018C	-
chrVI	198591	205460	CBS432_06G00790	YFR019W	+
chrVI	206940	207674	CBS432_06G00800	YFR020W	+
chrVI	210991	213186	CBS432_06G00820	YFR022W	+
chrVI	222152	223810	CBS432_06G00870	YFR028C	-
chrVI	224631	226670	CBS432_06G00880	YFR029W	+
chrVI	227019	230126	CBS432_06G00890	YFR030W	+
chrVI	230316	233828	CBS432_06G00900	YFR031C	-
chrVI	235829	236668	CBS432_06G00920	YFR032C	-
chrVIII	454296	456659	CBS432_08G02230	YHR182W	+
chrVIII	457050	458519	CBS432_08G02240	YHR183W	+
chrVIII	458739	460544	CBS432_08G02250	YHR184W	+
chrVIII	462073	466752	CBS432_08G02270	YHR186C	-
chrIX	288915	292883	CBS432_09G01280	YIL030C	-
chrXII	765132	766070	CBS432_12G03530	YLR340W	+
chrXII	766678	768105	CBS432_12G03540	YLR341W	+

chrXII	819447	821420	CBS432_12G03800	YLR369W	+
chrXIII	460149	461858	CBS432_13G02210	YMR105C	-
chrXIII	462554	464434	CBS432_13G02220	YMR106C	-
chrXIII	471553	475206	CBS432_13G02250	YMR109W	+
chrXIII	475408	477006	CBS432_13G02260	YMR110C	-
chrXIII	477438	478817	CBS432_13G02270	YMR111C	-
chrXIII	480026	481309	CBS432_13G02290	YMR113W	+
chrXIII	482724	484229	CBS432_13G02310	YMR115W	+
chrXIII	486297	486938	CBS432_13G02330	YMR117C	-
chrXIII	487205	487795	CBS432_13G02340	YMR118C	-
chrXIII	489600	491495	CBS432_13G02350	YMR119W	+
chrXV	80252	81997	CBS432_15G00340	YOL122C	-
chrXVI	64736	67393	CBS432_16G00290	YPL248C	-
chrXVI	61679	64375	CBS432_16G00280	YPL249C	-
chrXVI	60719	61255	CBS432_16G00270	YPL249C-A	-
chrXVI	884690	887314	CBS432_16G04360	YPR194C	-
chrXVI	891881	893293	CBS432_16G04370	YPR196W	+
chrXVI	894183	895814	CBS432_16G04380	YPR198W	+

Gene coordinates from: Yue et al.[3]

Supplementary Table 3. GO enrichment using GOrilla[6] of the 51 fixed and introgressed genes in *SpC (*SpB*-like).**

GO term	Description	P-value	FDR p-value[7]	Genes
GO:0043200	Response to amino acid	9.51E-5	4.94E-01	KOG1 - **K**ontroler **O**f **G**rwoth<br>ASI1 - **A**mino acid **S**ensor-**I**ndependent<br>PTR3 - **P**eptide **TR**ansport
GO:0005978	Glycogen biosynthetic process	2.22E-4	5.77E-1	IGD1 - **I**nhibitor of **G**lycogen **D**ebranching<br>GSY1 - **G**lycogen **SY**nthase<br>PGM2 - **P**hospho**G**luco**M**utase

Supplementary Table 4: Nine fixed and introgressed genes in *SpD* that were transmitted from *SpB* to *SpC to *SpD*. These genes occur at fixed introgressed sites in *SpC** and were used for dating the age of the *SpD* lineage.**

Chromosome	GeneStart [bp position]	GeneEnd [bp position]	GeneID	GeneName	Strand
chrV	162251	163807	CBS432_05G00760	YER007W	+
chrV	165581	169597	CBS432_05G00780	YER008C	-
chrVI	182464	183885	CBS432_06G00720	YFR012W	+
chrVI	184488	186863	CBS432_06G00730	YFR013W	+
chrVI	187123	188460	CBS432_06G00740	YFR014C	-
chrVI	224631	226670	CBS432_06G00880	YFR029W	+
chrVI	227019	230126	CBS432_06G00890	YFR030W	+
chrVI	230316	233828	CBS432_06G00900	YFR031C	-
chrXII	819447	821420	CBS432_12G03800	YLR369W	+

Gene coordinates from: Yue et al.[3]

Supplementary Table 5: Subset of 66 strains from the 5 lineages in *S. paradoxus* used for the analysis in BEAST[8].

Strain Name*	Systematic Name	Lineage
14_045A	LL2014_045	*SpA*
14_055A	LL2014_055	*SpA*
16_119A	LL2016_119	*SpA*
16_190A	LL2016_190	*SpA*
16_296A	LL2016_296	*SpA*
LL2012-026A	LL2012-026	*SpA*
yHKS172A	yHKS172	*SpA*
yHRM71-2A	yHRM71-2	*SpA*
16_097B	LL2016_097	*SpB*
16_234B	LL2016_234	*SpB*
16_245B	LL2016_245	*SpB*
16_250B	LL2016_250	*SpB*
95-7-1DB	95-7-1D	*SpB*
LL13-049B	LL2013-049	*SpB*
LL2012_002B	LL2012_002	*SpB*
MSH-483B	MSH-483	*SpB*
R11B	R11	*SpB*
R42B	R42	*SpB*
UWOPS-79-140B	UWOPS-79-14	*SpB*
WX36B	WX36	*SpB*
WX7B	WX7	*SpB*
yHBJ9B	yHBJ9	*SpB*
yHKS223B	yHKS223	*SpB*
yHKS306B	yHKS306	*SpB*
YPS618B	YPS618	*SpB*
YPS631B	YPS631	*SpB*
14_100C	LL2014_100	*SpC*
14_124C	LL2014_124	*SpC*
16_035C	LL2016_035	*SpC*
LL13-152C	LL2013-152	*SpC*
LL17-2C	LL2017-2	*SpC*
LL2012-027C	LL2012-027	*SpC*
MSH2B12C	MSH2B12	*SpC*
yHKS225C	yHKS225	*SpC*
14_067Ci	LL2014_067	*SpC**
14_092Ci	LL2014_092	*SpC**
14_121Ci	LL2014_121	*SpC**

14_161Ci	LL2014_161	*SpC**
14_178Ci	LL2014_178	*SpC**
16_199Ci	LL2016_199	*SpC**
LL13-010Ci	LL2013-010	*SpC**
LL13-012Ci	LL2013-012	*SpC**
LL2011-005Ci	LL2011-005	*SpC**
LL2011_006Ci	LL2011_006	*SpC**
LL2012_016Ci	LL2012_016	*SpC**
LL2012-016Ci	LL2012-016	*SpC**
LL2012_018Ci	LL2012_018	*SpC**
LL2012-018Ci	LL2012-018	*SpC**
LL2012_020Ci	LL2012_020	*SpC**
PPC23-2Ci	PPC23-2	*SpC**
UCD62-186Ci	UCD62-186	*SpC**
UCD62-268Ci	UCD62-268	*SpC**
yHKS414Ci	yHKS414	*SpC**
B3D	B3	*SpD*
M1D	M1	*SpD*
M2D	M2	*SpD*
M3D	M3	*SpD*
R19D	R19	*SpD*
R20D	R20	*SpD*
R21D	R21	*SpD*
R22D	R22	*SpD*
R23D	R23	*SpD*
R24D	R24	*SpD*
WX19D	WX19	*SpD*
WX20D	WX20	*SpD*
WX21D	WX21	*SpD*

Note that to simplify the discrimination of strains, some might have a lineage-identifier in the end of its name (e.g. "B" for SpB, "D" for SpD or "Ci" for SpC)

Supplementary Table 6: Results of dating the divergence of *S. paradoxus* lineages.

Divergence between:	9 genes (21 kb)	4 fragments (630 kb)	Leducq et al.[1]
SpA from *SpB-SpC*	158,000 ± 78,000 years ago	139,000 ± 28,000 years ago	176,200 ± 36,600 years ago
SpB and *SpC*	96,000 ± 20,000 years ago	96 ± 20,000 years ago	110,000 ± 10,000 years ago
SpB and *SpC**	18,500 ± 5,000 years ago	Not calculated	15,400 ± 4,000 years ago
SpC and *SpC**	Not calculated[i]	Not calculated	10,500 ± 4,300 years ago
*SpC** and *SpD*	2,600 ± 950 years ago	4,100 ± 850 years ago	No data available
**Divergence within:**			
SpA	230 ± 70 years ago	300 ± 100 years ago	286 ± 174 years ago
SpB	14,800 ± 3,900 (34,700 ± 8,500[ii]) years ago	18,100 ± 3,700 (34 ± 7,000[ii]) years ago	15,400 ± 4,000 years ago
SpC	6,800 ± 2,500 years ago	Not calculated	8,200 ± 2,300 years ago
*SpC**	4,000 ± 1,450 years ago	Not calculated	6,500 to 10,400 years ago
SpD	1,800 ± 750 years ago	3,000 ± 600 years ago	No data available

[i] Not available, as *SpC* and *SpC** do not share direct inheritance for introgressed genes of *SpB*-origin

[ii] including outlier yHKS306B that branches earlier

Supplementary Table 7. List of strains as part of the transcriptomic study.

Systematic Name	Mating_type	Genotype	Genetic_Group	Location_Sampled
95-7-1D	Diploid	Wild	*SpB*	East Lansing, Michigan, USA
LL2011_003	Diploid	Wild	*SpC*	Cap Chat, Quebec, Canada
LL2011_005	Diploid	Wild	*SpC**	Ile d'Orleans, Quebec, Canada
LL2011_006	Diploid	Wild	*SpC**	Ile d'Orleans, Quebec, Canada
LL2012_014	Diploid	Wild	*SpB*	St Michel de Bellechasse, Quebec, Canada
LL2012_016	Diploid	Wild	*SpC**	Pointe Platon, Quebec, Canada
LL2012_016	Diploid	Wild	*SpC**	Pointe Platon, Quebec, Canada
LL2012_020	Diploid	Wild	*SpC**	Pointe Platon, Quebec, Canada
LL2012_026	Diploid	Wild	*SpA*	Sherbrooke, Quebec, Canada
LL2012_027	Diploid	Wild	*SpC*	Sherbrooke, Quebec, Canada
LL2013_012	Diploid	Wild	*SpC**	Quebec, Quebec, Canada
MSH-587-1	Diploid	Wild	*SpC*	Mont St Hilaire, Quebec, Canada
MSH-604	Diploid	Wild	*SpB*	Mont St Hilaire, Quebec, Canada
R13	Diploid	Wild	*SpB*	University of Toronto Mississauga, Ontario, Canada
R19	Diploid	Wild	*SpD*	University of Toronto Mississauga, Ontario, Canada
R20	Diploid	Wild	*SpD*	University of Toronto Mississauga, Ontario, Canada
R22	Diploid	Wild	*SpD*	University of Toronto Mississauga, Ontario, Canada
R24	Diploid	Wild	*SpD*	University of Toronto Mississauga, Ontario, Canada
R33	Diploid	Wild	*SpB*	University of Toronto Mississauga, Ontario, Canada
UWOPS_79-140	Diploid	Wild	*SpB*	Saint-Joseph Island, Ontario, Canada
WX19	Diploid	Wild	*SpD*	University of Toronto Mississauga, Ontario, Canada
WX21	Diploid	Wild	*SpD*	University of Toronto Mississauga, Ontario, Canada
WX26	Diploid	Wild	*SpB*	University of Toronto Mississauga, Ontario, Canada
YPS744	Diploid	Wild	*SpA*	Tuscarora, New York, USA

Supplementary Table 7 (continued).

Systematic Name	Substrate_Sampled	Published in	lon	lat
95-7-1D	Bark - Oak tree	Leducq, et al. [1]	-84,4838654	42,7369792
LL2011_003	Bark - Mapple tree	Leducq, et al. [1]	-66,6857394	49,0940662
LL2011_005	Bark - Mapple tree	Leducq, et al. [1]	-70,931194	46,9295317
LL2011_006	Rotten apple	Leducq, et al. [1]	-66,6857394	49,0940662
LL2012_014	Bark - Mapple tree	Leducq, et al. [1]	-70,9095727	46,8554265
LL2012_016	Bark - Oak tree	Leducq, et al. [1]	-71,859351	46,665643
LL2012_016	Bark - Oak tree	Leducq, et al. [1]	-71,859351	46,665643
LL2012_020	Bark - Oak tree	Leducq, et al. [1]	-71,859351	46,665643
LL2012_026	Bark - Mapple tree	Leducq, et al. [1]	-71,8824288	45,4009928
LL2012_027	Bark - Mapple tree	Leducq, et al. [1]	-71,8824288	45,4009928
LL2013_012	Soil - Oak tree	Leducq, et al. [1]	-71,2079809	46,8138783
MSH-587-1	Bark - Oak tree	Leducq, et al. [1]	-73,1790126	45,5640416
MSH-604	Bark - Oak tree	Leducq, et al. [1]	-73,1790126	45,5640416
R13	Quercus	Xia, et al. [2]	-79,6660259	43,5471097
R19	Quercus	Xia, et al. [2]	-79,6660259	43,5471097
R20	Quercus	Xia, et al. [2]	-79,6660259	43,5471097
R22	Quercus	Xia, et al. [2]	-79,6660259	43,5471097
R24	Quercus	Xia, et al. [2]	-79,6660259	43,5471097
R33	Quercus	Xia, et al. [2]	-79,6660259	43,5471097
UWOPS_79-140	Knot - Apple tree	Leducq, et al. [1]	-83,9667213	46,2227098
WX19	Quercus	Xia, et al. [2]	-79,6660259	43,5471097
WX21	Quercus	Xia, et al. [2]	-79,6660259	43,5471097
WX26	Quercus	Xia, et al. [2]	-79,6660259	43,5471097
YPS744	Bark - Oak tree	Leducq, et al. [1]	-77,8697236	42,6306196

Supplementary Table 8: Assembly and annotation statistics of lineage-specific reference genomes for the transcriptomic analysis.

Assembly:

**Lineage**	Total sequence length	Number of contigs	Contigs N50	Number of chromosomes	Total number of scaffolds*
SpA	11622321	781	139392	17	85
SpB	11736119	706	154133	17	73
SpC	11789292	830	122691	17	68
*SpC**	11786280	773	131537	17	17
SpD1	12012101	1297	124328	17	227
SpD2	11927420	1323	153967	17	128

Annotation:

**Lineage**	Augustus[9] gene prediction	Extended prediction**	Annotated genes***
SpA	5398	6636	5316
SpB	5409	6692	5335
SpC	5411	6868	5315
*SpC**	5427	6778	5339
SpD1	5438	6965	5355
SpD2	5435	6995	5350

* chromosomes + unplaced scaffolds

** from transcription data

*** detected orthologous gene in reference strain CBS432

SUPPLEMENTARY REFERENCES

1 Leducq, J.-B. *et al.* Speciation driven by hybridization and chromosomal plasticity in a wild yeast. *Nature Microbiology* **1**, 15003, doi:10.1038/nmicrobiol.2015.3 (2016).

2 Xia, W. *et al.* Population genomics reveals structure at the individual, host-tree scale and persistence of genotypic variants of the undomesticated yeast Saccharomyces paradoxus in a natural woodland. *Mol Ecol* **26**, 995-1007, doi:10.1111/mec.13954 (2017).

3 Yue, J. X. *et al.* Contrasting evolutionary genome dynamics between domesticated and wild yeasts. *Nat Genet* **49**, 913-924, doi:10.1038/ng.3847 (2017).

4 Earl, D. A. & vonHoldt, B. M. STRUCTURE HARVESTER: a website and program for visualizing STRUCTURE output and implementing the Evanno method. *Conservation Genetics Resources* **4**, 359-361, doi:10.1007/s12686-011-9548-7 (2012).

5 Love, M. I., Huber, W. & Anders, S. Moderated estimation of fold change and dispersion for RNA-seq data with DESeq2. *Genome Biol* **15**, 550, doi:10.1186/s13059-014-0550-8 (2014).

6 Eden, E., Navon, R., Steinfeld, I., Lipson, D. & Yakhini, Z. GOrilla: a tool for discovery and visualization of enriched GO terms in ranked gene lists. *BMC Bioinformatics* **10**, 48, doi:10.1186/1471-2105-10-48 (2009).

7 Benjamini, Y. & Hochberg, Y. Controlling the False Discovery Rate: A Practical and Powerful Approach to Multiple Testing. *Journal of the Royal Statistical Society. Series B (Methodological)* **57**, 289-300 (1995).

8 Bouckaert, R. *et al.* BEAST 2: a software platform for Bayesian evolutionary analysis. *PLoS Comput Biol* **10**, e1003537, doi:10.1371/journal.pcbi.1003537 (2014).

9 Stanke, M. *et al.* AUGUSTUS: ab initio prediction of alternative transcripts. *Nucleic Acids Res* **34**, W435-439, doi:10.1093/nar/gkl200 (2006).
