# Supplementary material for: Hybridization is a recurrent evolutionary stimulus in wild yeast speciation
Source: Nat Commun. 2019 Feb 25;10:923. doi: 10.1038/s41467-019-08809-7 (PMC6389940; doi:10.1038/s41467-019-08809-7)
Supplement: Supplementary file 3 — Description of Additional Supplementary Files [file 41467_2019_8809_MOESM3_ESM.pdf]

**Description of Additional Supplementary Files**

File Name: Supplementary Data 1
Description: Geographical location of strains from 5 groups in North America.

File Name: Supplementary Data 2
Description: Sequence statistics for 74 newly sequenced genomes with Illumina.

File Name: Supplementary Data 3
Description: Twenty-five conditions used for high-throughput growth measurement.

File Name: Supplementary Data 4
Description: Library and sequencing statistics for the 48 transcriptomes.

File Name: Supplementary Data 5
Description: GO enrichment using GOrilla of pairwise comparisons with ranked gene lists using P-adjust.

File Name: Supplementary Data 6
Description: Reproductive isolation from spore viability of intra- and inter-lineage crosses.
