# Supplementary material for: Hybridization is a recurrent evolutionary stimulus in wild yeast speciation
Source: Nat Commun. 2019 Feb 25;10:923. doi: 10.1038/s41467-019-08809-7 (PMC6389940; doi:10.1038/s41467-019-08809-7)
Supplement: Supplementary file 10 — Reporting Summary [file 41467_2019_8809_MOESM10_ESM.pdf]

nature research

Corresponding author(s): Dr. Christian Landry
Dr. Chris Eberlein

Last updated by author(s): January 11th, 2019

Reporting Summary

Nature Research wishes to improve the reproducibility of the work that we publish. This form provides structure for consistency and transparency in reporting. For further information on Nature Research policies, see Authors & Referees and the Editorial Policy Checklist.

Statistics

For all statistical analyses, confirm that the following items are present in the figure legend, table legend, main text, or Methods section.

n/a	Confirmed	
☐	☒	The exact sample size (n) for each experimental group/condition, given as a discrete number and unit of measurement
☐	☒	A statement on whether measurements were taken from distinct samples or whether the same sample was measured repeatedly
☐	☒	The statistical test(s) used AND whether they are one- or two-sided<br>*Only common tests should be described solely by name; describe more complex techniques in the Methods section.*
☐	☒	A description of all covariates tested
☐	☒	A description of any assumptions or corrections, such as tests of normality and adjustment for multiple comparisons
☐	☒	A full description of the statistical parameters including central tendency (e.g. means) or other basic estimates (e.g. regression coefficient) AND variation (e.g. standard deviation) or associated estimates of uncertainty (e.g. confidence intervals)
☐	☒	For null hypothesis testing, the test statistic (e.g. F, t, r) with confidence intervals, effect sizes, degrees of freedom and P value noted<br>*Give P values as exact values whenever suitable.*
☐	☒	For Bayesian analysis, information on the choice of priors and Markov chain Monte Carlo settings
☐	☒	For hierarchical and complex designs, identification of the appropriate level for tests and full reporting of outcomes
☐	☒	Estimates of effect sizes (e.g. Cohen's d, Pearson's r), indicating how they were calculated

Our web collection on statistics for biologists contains articles on many of the points above.

Software and code

Policy information about availability of computer code

Data collection	The programs used for data collection and their version(s) are mentioned where relevant in the method/result sections.
Data analysis	The programs used for analysis and their version(s) are mentioned where relevant in the method/result sections. Any custom code can be shared upon request.

For manuscripts utilizing custom algorithms or software that are central to the research but not yet described in published literature, software must be made available to editors/reviewers. We strongly encourage code deposition in a community repository (e.g. GitHub). See the Nature Research guidelines for submitting code & software for further information.

Data

Policy information about availability of data

All manuscripts must include a data availability statement. This statement should provide the following information, where applicable:

- Accession codes, unique identifiers, or web links for publicly available datasets
- A list of figures that have associated raw data
- A description of any restrictions on data availability

The data that support the findings of this study are available from the corresponding authors upon request.

Field-specific reporting

Please select the one below that is the best fit for your research. If you are not sure, read the appropriate sections before making your selection.

☐ Life sciences ☐ Behavioural & social sciences ☒ Ecological, evolutionary & environmental sciences

Ecological, evolutionary & environmental sciences study design

All studies must disclose on these points even when the disclosure is negative.

Study description	This study investigates the recurrent hybridization of naturally occurring yeast Saccharomyces paradoxus in North America. We studied the genetic and phenotypic fingerprints of this hybridization event. The population study is based on 316 whole genome sequences in total, including 72 newly whole-genome sequenced strains (Illumina), 6 de novo genome assemblies (sequence with Oxford Nanopore technology) and 46 transcriptomes. High-throughput colony growth measurement was done using 25 culture conditions and measured through time.
Research sample	The newly sequenced strains of Saccharomyces paradoxus were isolated between 2014 and 2016 from the bark of trees or the soil associated with trees in North America. The collection of samples used in the population genomics analysis (total 316 strains from 5 different genetic lineages) is meant to represent the population diversity on these types of substrates in North America.
Sampling strategy	We used the protocol as described in the research articles below to isolate and select strains:<br>Sniegowski, P. D., Dombrowski, P. G. & Fingerman, E. Saccharomyces cerevisiae and Saccharomyces paradoxus coexist in a natural woodland site in North America and display different levels of reproductive isolation from European conspecifics. FEMS Yeast Res 1, 299-306 (2002).<br>Leducq, J. B. et al. Local climatic adaptation in a widespread microorganism. Proc Biol Sci 281, 20132472, doi:10.1098/rspb.2013.2472 (2014).
Data collection	Sampling of the strains was done by Guillaume Charron, Mathieu Henault, Jean-Baptiste Leducq, James B Anderson, Anne-Marie Dion-Côté, Andrew Clark, Sarah Lower, Ian Caldas, Yasir Ahmed and Sofie Delbare.<br>Short read sequencing data was collected by MH and CE using standard protocols made available by the manufacturer.<br>Long read sequencing data was collected by MH using standard protocols made available by the manufacturer.<br>Colony growth data was collected by MH and CE using a robotic platform for yeast colony manipulation and image acquisition.<br>RNA-seq data was collected by CE using standard protocols made available by the manufacturer.<br>New fertility data was collected by mating strains from the different groups and sporulation of the resulting hybrids following standard protocols. For each hybrid, 96 tetrads were dissected and the fertility was recorded by GC.
Timing and spatial scale	Sampling of the new strains published in this study was done from 2014 to 2016.<br>Short read whole-genome sequencing data collection was done in August 2017.<br>Long read whole-genome sequencing data collection was done from September to October 2017.<br>Colony growth data collection took place in February 2018.<br>RNA-seq data collection was done May and September 2017.<br>Fertility Data was collected in March 2018.
Data exclusions	We initially sequenced 74 strains (whole genome sequencing) and we removed 2 (see suppl. material) because of 1 contamination and 1 strain with too low coverage.<br>One culture condition (synthetic medium with lysine) was excluded from the colony growth analysis because of uniformly poor growth of the strains in it, yielding low and noisy growth signal. One strain was excluded due to visible contamination. Three strains were excluded because at least one of their growth metrics was null in at least one condition. Eight strains were excluded because of their overall poor growth on all conditions following a preliminary principal component analysis.<br>From the initial 48 transcriptomes, we removed 2 libraries (see suppl. material) because they showed extremely different gene expression signals in their biological replicates as well with other strains from the same genetic lineage.<br>No data was excluded from fertility analyses.
Reproducibility	For the colony growth experiment, 12 replicates of each strain were split on four different solid media plates, ensuring that the analysis results are robust with respect to experimental variation. For the RNA-seq experiment, two replicate sequencing libraries were prepared for each strain.
Randomization	For the colony growth experiment, the spatial position of each strain replicate on each solid media plate was randomly assigned to avoid colony growth effects due to intra-plate variation (ex. central position versus proximity to a plate border).
Blinding	Blinding was not relevant to this study because there was no use of human subjects.

Did the study involve field work? ☒ Yes ☐ No

Field work, collection and transport

Field conditions	The new isolated samples in this study were isolated between 2014 and 2016 during the summer months.
Location	The geographic coordinates for each sample are listed in the Supplementary Data.
Access and import/export	No permit was needed during sampling and transport of isolates.
Disturbance	We isolated samples of tree bark or soil associated with trees and samples were processed within the following 7 days.

Reporting for specific materials, systems and methods

We require information from authors about some types of materials, experimental systems and methods used in many studies. Here, indicate whether each material, system or method listed is relevant to your study. If you are not sure if a list item applies to your research, read the appropriate section before selecting a response.

Materials & experimental systems

n/a	Involved in the study
☒	☐ Antibodies
☒	☐ Eukaryotic cell lines
☒	☐ Palaeontology
☐	☒ Animals and other organisms
☒	☐ Human research participants
☒	☐ Clinical data

Methods

n/a	Involved in the study
☒	☐ ChIP-seq
☒	☐ Flow cytometry
☒	☐ MRI-based neuroimaging

Animals and other organisms

Policy information about studies involving animals; ARRIVE guidelines recommended for reporting animal research

Laboratory animals	The study did not involve laboratory animals.
Wild animals	The study does not involve wild animals. We sampled yeast strains from trees.
Field-collected samples	Yeast strains were sampled during summer time between the years 2014-2016.
Ethics oversight	No ethical approval or guidance was required since we sampled either soil or bark to isolate yeast strains.

Note that full information on the approval of the study protocol must also be provided in the manuscript.
