# Supplementary material for: Author Correction: Hybridization is a recurrent evolutionary stimulus in wild yeast speciation
Source: Nat Commun. 2019 May 13;10:2199. doi: 10.1038/s41467-019-09702-z (PMC6513852; doi:10.1038/s41467-019-09702-z)
Supplement: Supplementary file 1 — Supplementary Information [file 41467_2019_9702_MOESM1_ESM.pdf]

A

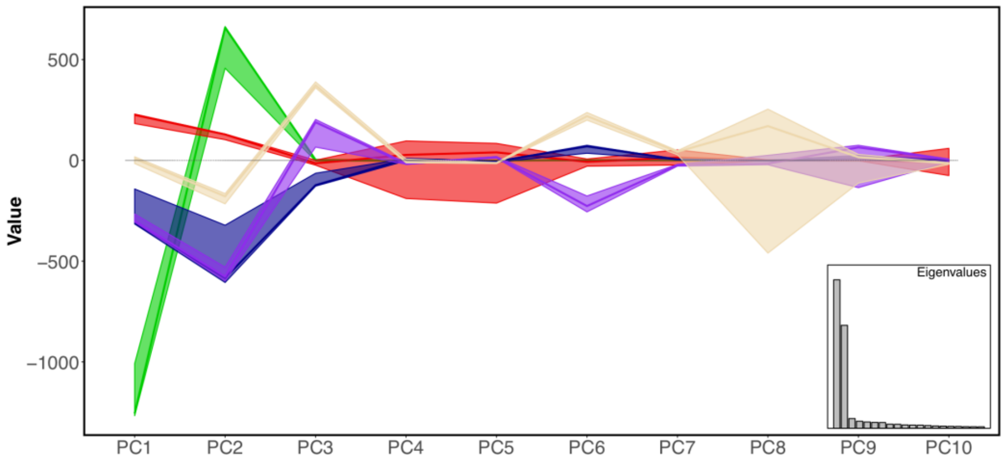

B

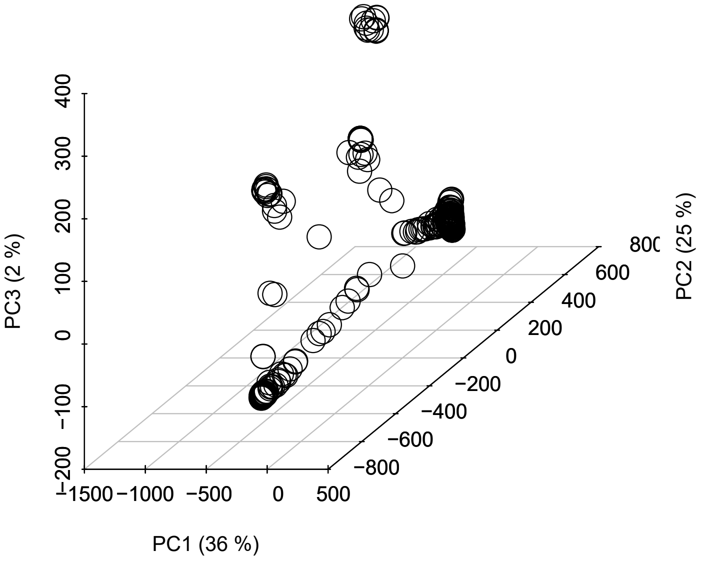

Supplementary Figure 2: Principal component analysis (PCA) from genome-wide SNP data distinguishes the 5 main *S. paradoxus* groups.

(A) A principal component analysis on 205,206 variants distributed genome-wide in 316 genomes (only showing the first ten PCs) was performed to examine the different *S. paradoxus* lineages. The bold line shows the median value for each lineage and shades the 15% and 85% quantiles. The lineages *SpC* and *SpC**, differentiate at PC3 and PC6. *SpD* clusters independently from the other lineages. **(B)** A 3D visualization of the first 3 PCs shows the separation into the 5 genetically different clusters. The colors correspond to the strains of the different lineages (green = *SpA*, red = *SpB*, blue = *SpC*, purple = *SpC**, beige = *SpD*).

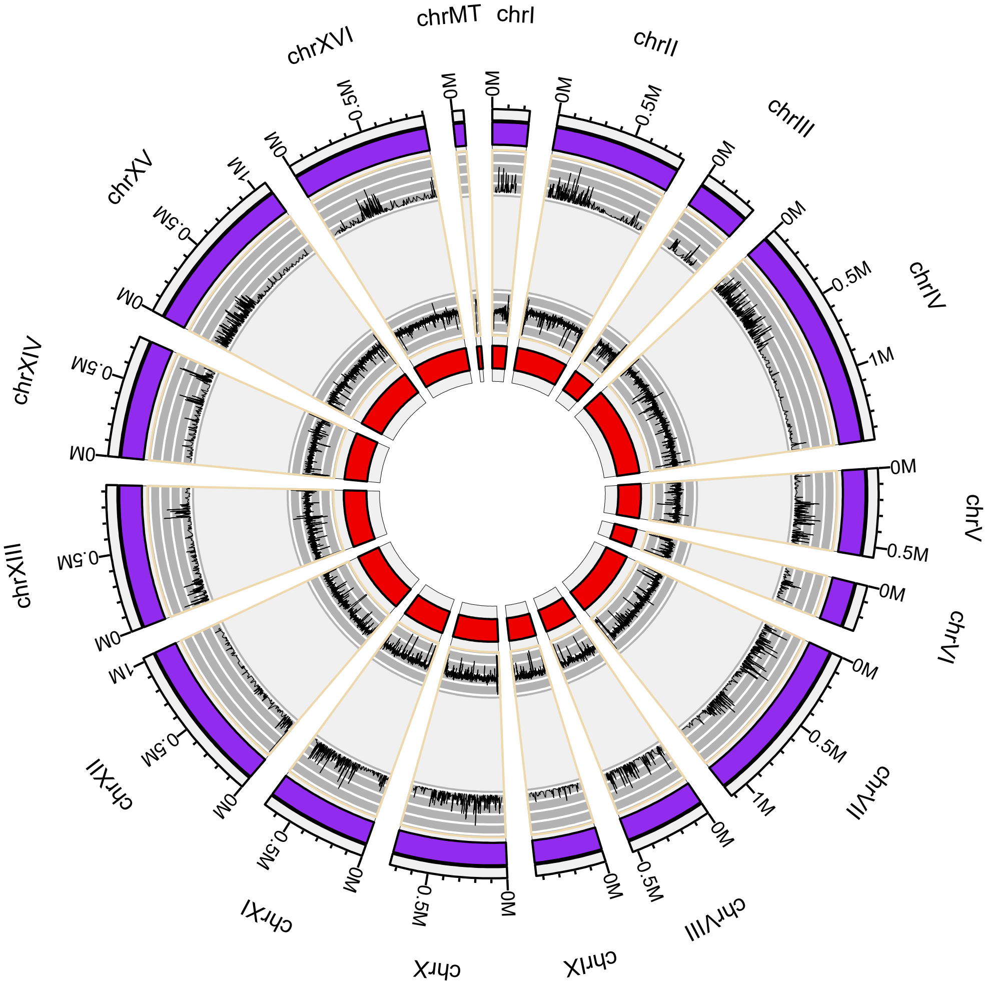

Supplementary Figure 12: *SpD* strain WX21 shows heterozygous loci across the genome.

The *SpD* strain WX21 shows many regions that could not be assigned confidently to *SpB* (inner red ring) or *SpC** (outer purple ring) introgression (blank regions, middle circle), which stands in contrast to the other 12 *SpD* strains (Figure 3A). We confirmed the presence of mapped sequence data (inner circle, coverage data corresponds to the average coverage from 1 kb windows) throughout the genome and could show that the non-defined regions (in white) correspond to peaks of heterozygosity (outer rings; counts of heterozygous sites were calculated per 1kb window).

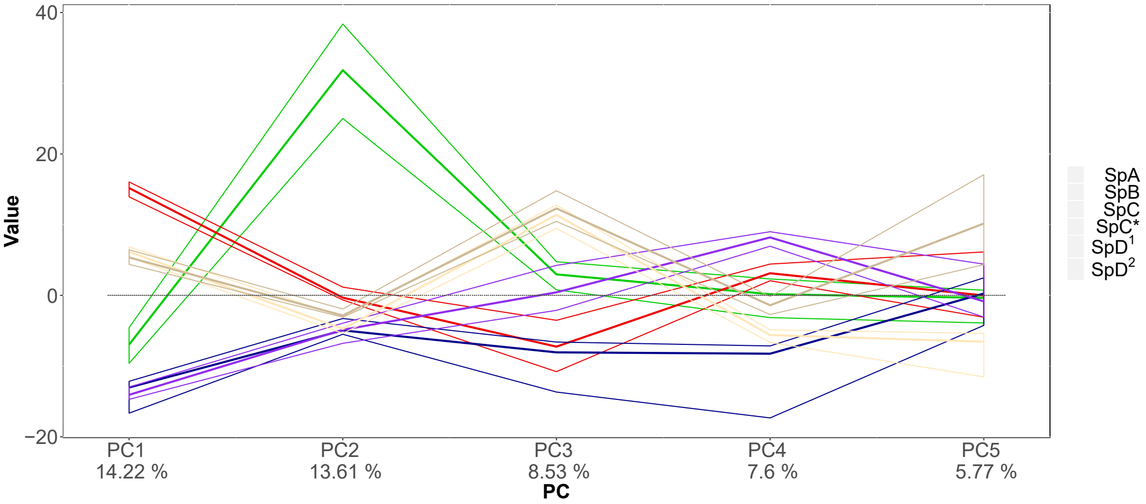

Supplementary Figure 20: Grouping of strains according to principal component analysis based on expression levels of 5,160 genes.

The most important PC, PC1, distinguishes the main groups *SpA*, *SpB*, *SpC* and *SpD* but does not distinguish *SpC** and *SpC*. PC3 and PC4 splits *SpC* and *SpC** strains into separate clusters, PC5 splits the two different *SpD* clades. Bold lines correspond to the median value and the color shades to the 15% and 85% quantiles.

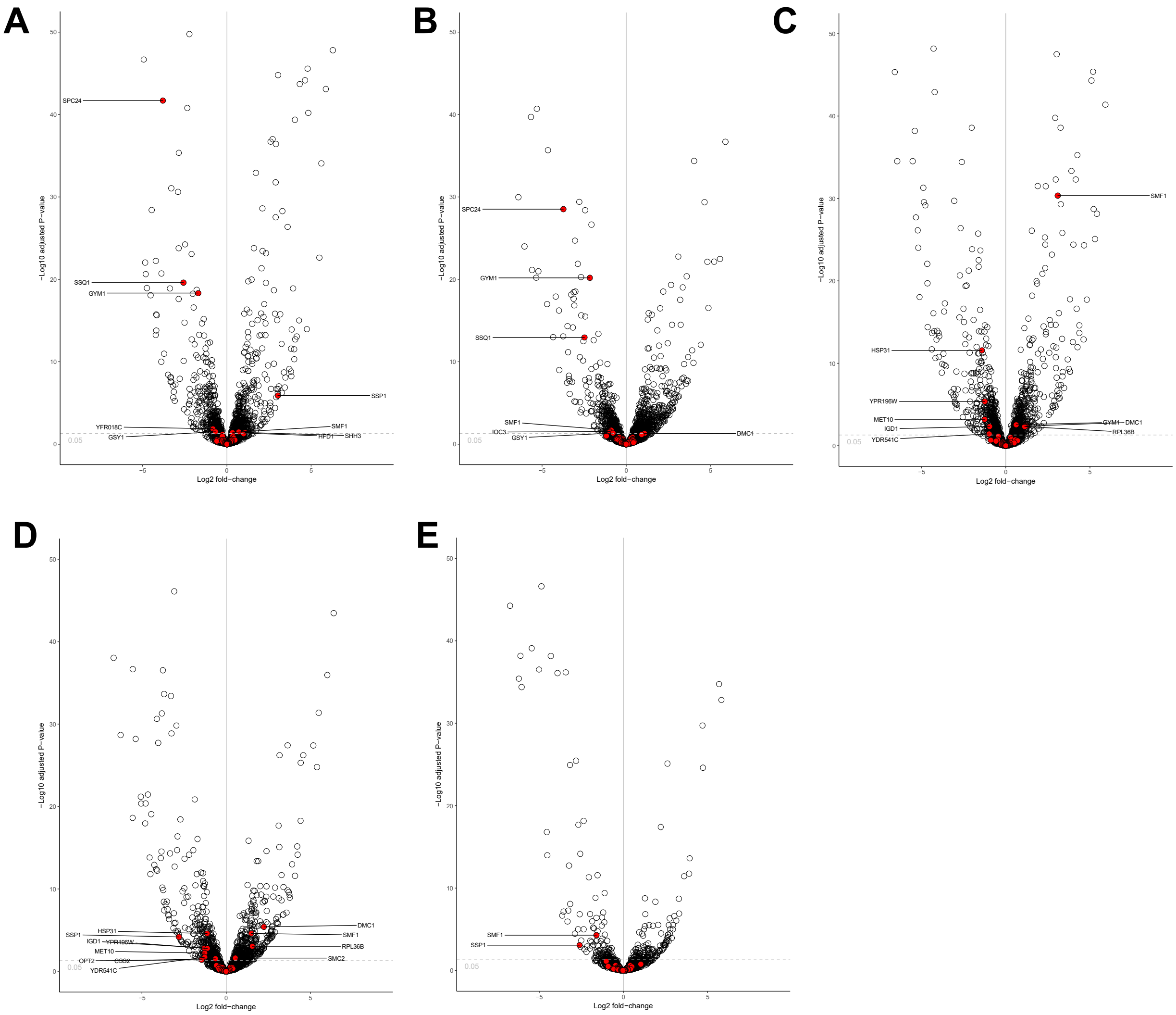

Supplementary Figure 22: Pairwise comparison of gene expression between the lineages *SpB*, *SpC*, *SpD1* and *SpD2*.

Pairwise comparison of **(A)** *SpB* and *SpD1,* **(B)** *SpB* and *SpD2,* **(C)** *SpC** and *SpD1,* **(D)** *SpC** and *SpD2,* and **(E)** *SpD1* and *SpD2.* Volcano plots showing the pairwise comparison of expression of 5,160 genes. We used the average expression per gene for each lineage to performed subsequent Gene-Ontology enrichment analyses on ranked data (p-adjust value; Supplementary table 7). Lineages are color coded (*SpB* = red, *SpC* = blue, *SpC** = purple, *SpD*= beige). In dark red are the expression profiles of 48 out of the 51 introgressed genes that are fixed in all *SpC** individuals and have been inherited from *SpB*.
